# Evidence for Aberrant Astrocyte Hemichannel Activity in Juvenile Neuronal Ceroid Lipofuscinosis (JNCL)

**DOI:** 10.1371/journal.pone.0095023

**Published:** 2014-04-15

**Authors:** Maria Burkovetskaya, Nikolay Karpuk, Juan Xiong, Megan Bosch, Michael D. Boska, Hideyuki Takeuchi, Akio Suzumura, Tammy Kielian

**Affiliations:** 1 Departments of Pathology and Microbiology, University of Nebraska Medical Center, Omaha, Nebraska, United States of America; 2 Departments of Pharmacology and Experimental Neuroscience, University of Nebraska Medical Center, Omaha, Nebraska, United States of America; 3 Department of Radiology, University of Nebraska Medical Center, Omaha, Nebraska, United States of America; 4 Department of Neuroimmunology, Research Institute of Environmental Medicine, Nagoya University, Nagoya, Japan; Kyushu University, Japan

## Abstract

Juvenile Neuronal Ceroid Lipofuscinosis (JNCL) is a lysosomal storage disease caused by an autosomal recessive mutation in *CLN3* that leads to vision loss, progressive cognitive and motor decline, and premature death. Morphological evidence of astrocyte activation occurs early in the disease process and coincides with regions where neuronal loss eventually ensues. However, the consequences of CLN3 mutation on astrocyte function remain relatively ill-defined. Astrocytes play a critical role in CNS homeostasis, in part, by their ability to regulate the extracellular milieu via the formation of extensive syncytial networks coupled by gap junction (GJ) channels. In contrast, unopposed hemichannels (HCs) have been implicated in CNS pathology by allowing the non-discriminant passage of molecules between the intracellular and extracellular milieus. Here we examined acute brain slices from CLN3 mutant mice (CLN3^Δex7/8^) to determine whether CLN3 loss alters the balance of GJ and HC activity. CLN3^Δex7/8^ mice displayed transient increases in astrocyte HC opening at postnatal day 30 in numerous brain regions, compared to wild type (WT) animals; however, HC activity steadily decreased at postnatal days 60 and 90 in CLN3^Δex7/8^ astrocytes to reach levels lower than WT cells. This suggested a progressive decline in astrocyte function, which was supported by significant reductions in glutamine synthetase, GLAST, and connexin expression in CLN3^Δex7/8^ mice compared to WT animals. Based on the early increase in astrocyte HC activity, CLN3^Δex7/8^ mice were treated with the novel carbenoxolone derivative INI-0602 to inhibit HCs. Administration of INI-0602 for a one month period significantly reduced lysosomal ceroid inclusions in the brains of CLN3^Δex7/8^ mice compared to WT animals, which coincided with significant increases in astrocyte GJ communication and normalization of astrocyte resting membrane potential to WT levels. Collectively, these findings suggest that alterations in astrocyte communication may impact the progression of JNCL and could offer a potential therapeutic target.

## Introduction

Juvenile Neuronal Ceroid Lipofuscinosis (JNCL), or Juvenile Batten Disease, is an autosomal recessively inherited lysosomal storage disorder caused by mutations in the *CLN3* gene [1]. In general, children with JNCL develop neurological symptoms beginning at 5–8 years of age typified by vision loss, behavioral disturbances, and seizure activity. The disease is associated with progressive neurological decline, involving substantial motor and cognitive loss and premature death by the late-teens to early 20 s [2], [3]. The CNS is particularly vulnerable in JNCL, although systemic complications are also observed, since inclusions form in multiple cell types in the body [4]. In the CNS, neuronal loss is more pronounced in specific thalamocortical structures, including the thalamic nuclei, neocortex, substantia nigra, hippocampus, and cerebellum [5], [6], [7], [8]. CLN3 mutation leads to the progressive accumulation of autofluorescent ceroid inclusions in the lysosome, which are predominantly composed of mitochondrial ATP synthase subunit c [9], [10]. Interestingly, similar inclusions can occur in the aged brain, commonly referred to as lipofuscin, which can be detected in Alzheimer’s or Parkinson’s disease patients [11], [12], [13], [14]. This suggests the possibility of common underlying pathologies between these neurodegenerative disorders, and although JNCL presents within the first decade of life, it is notable that these children progress to develop Parkinson-like symptoms that coincide with neuronal loss in the substantia nigra [15]. Indeed, evidence is emerging suggesting conserved autophagy and mitochondrial abnormalities associated with NCLs and adult-onset neurodegenerative diseases [16], [17]. This suggests that studies investigating mechanisms of CNS dysfunction during JNCL may also unveil novel pathways common to other neurodegenerative disorders.

Astrocytes and microglia are key contributors to neuronal homeostasis and function [18], [19]. Prior studies using CLN3 knockout mice or animals where exons 7 and 8 of the *CLN3* gene were disrupted (CLN3^Δex7/8^), demonstrated early signs of glial activation that preceded neuronal loss [8], [20]. Specifically, morphological evidence of glial activation was apparent by postnatal day 7; however, neuronal death was significantly delayed in comparison (i.e. apparent around 6–8 months). These findings suggest that chronic glial activation may provide extrinsic signals that influence neuronal survival at later disease intervals, although intrinsic defects in neurons cannot be ignored. Indeed, we recently reported that primary microglia from CLN3^Δex7/8^ mice are primed towards a proinflammatory phenotype and secrete heightened levels of numerous inflammatory mediators following exposure to stimuli that are elevated in the JNCL brain [21]. In addition, CLN3^Δex7/8^ microglia displayed constitutive caspase-1 activity, which when inhibited resulted in enhanced glutamate release via hemichannel action that induced CLN3^Δex7/8^ neuron death [21]. In contrast, limited information is currently available concerning the functional implications of CLN3 mutation in astrocytes. Since astrocytes play a central role in maintaining CNS homeostasis [22], [23], glucose availability [24], and neurotransmitter utilization, pathological alterations in astrocyte activity in the context of CLN3 mutation may contribute to neuronal death during JNCL. If correct, identifying ways to reverse astrocyte dysfunction to reinstate normal attributes may promote neuronal survival.

Hemichannels (HCs) are non-selective pores located predominantly in the plasma membrane, which permit the free passage of various ions and small organic molecules (<1.5 kDa) between the extracellular and intracellular milieus. Although still an area of debate, numerous reports have revealed that astrocyte HCs are composed of connexin 43 (Cx43), whereas others have described pannexin involvement [25], [26]. Two Cx HCs from neighboring astrocytes can interact to form a gap junction (GJ) channel. Numerous GJ channels organize astrocytes into broad syncytial networks, facilitating the rapid exchange of intracellular contents in a process referred to as GJ communication (GJC) [27], [28], [29], [30], [31]. Astrocyte GJC plays a role in the homeostatic regulation of extracellular pH, K^+^, and glutamate levels [32], [33], [34]. Astrocytes also influence CNS vascular tone and neuronal synapses, which are facilitated, in part, via GJC [35], [36], [37], [38]. Therefore, the balance between astrocyte GJC versus HC activity in the context of CLN3 mutation could influence the homeostatic balance of the CNS milieu and impact neuronal viability. Here we investigated the status of astrocyte GJC/HC activity and associated intrinsic electrophysiological properties using acute brain slices from CLN3^Δex7/8^ mice at three postnatal stages that significantly precede neuronal loss [8], [20]. Postnatal day 30 was selected as a starting point for our experiments, since this generally extrapolates to an age when a positive diagnosis of JNCL is made in children [39], [40], with the eventual goal of identifying abnormalities that could be targeted to delay/prevent neuronal loss during later stages of JNCL. This study is the first to report astrocyte communication and electrophysiological defects in the context of JNCL and importantly, our use of living brain slices provides an excellent model to assess the interplay between astrocytes and other CNS cell types.

Previous work by us and others has demonstrated that astrocyte HC activity is enhanced during neuroinflammatory conditions [41], [42], [43]. Since other forms of Batten Disease have been associated with neuroinflammation, the severity of which depends on the specific disease type [44], [45], [46], [47], we were interested in examining whether astrocyte HC activity was modulated in JNCL. Here we report that CLN3^Δex7/8^ astrocytes in acute brain slices displayed increased HC activity in the majority of brain regions examined at postnatal day 30, which coincided with altered electrophysiological properties. However, these changes were transient, in that CLN3^Δex7/8^ astrocyte HC activity gradually decreased between postnatal days 60 and 90, and in some instances was lower than astrocytes from WT brain slices, suggesting a progressive deterioration in CLN3^Δex7/8^ astrocyte activity. Evidence to support a decline in astrocyte function was demonstrated by the fact that several molecules associated with glutamate homeostasis (i.e. glutamine synthetase and the glutamate-aspartate transporter GLAST) were significantly decreased in CLN3^Δex7/8^ mice at postnatal day 90. In contrast, GFAP expression was significantly elevated in several brain regions of CLN3^Δex7/8^ animals, in agreement with previous reports [8], [20], indicating that the decreases in GLAST and glutamine synthetase detected in CLN3^Δex7/8^ mice is not the result of astrocyte loss. Rather, these findings reveal the attrition of molecules that regulate glutamate homeostasis, which likely triggers astrocyte activation in an attempt to rectify this decline. Treatment of CLN3^Δex7/8^ mice with the carbenoxolone (CBX) derivative INI-0602 to block elevated HC activity at postnatal day 30, led to improvements in several pathological changes typical of JNCL, including significant reductions in lysosomal ceroid inclusions within the CNS, which coincided with enhanced GJC. Collectively, these results suggest that astrocyte dysfunction is evident during early JNCL and that modulation of GJC/HC activity may represent a promising target to reverse some pathological outcomes typical of the disease.

## Results

### Use of CellTracker Blue (CTB) to Visualize Live Astrocytes in Acute Brain Slices

**Figure 1:**
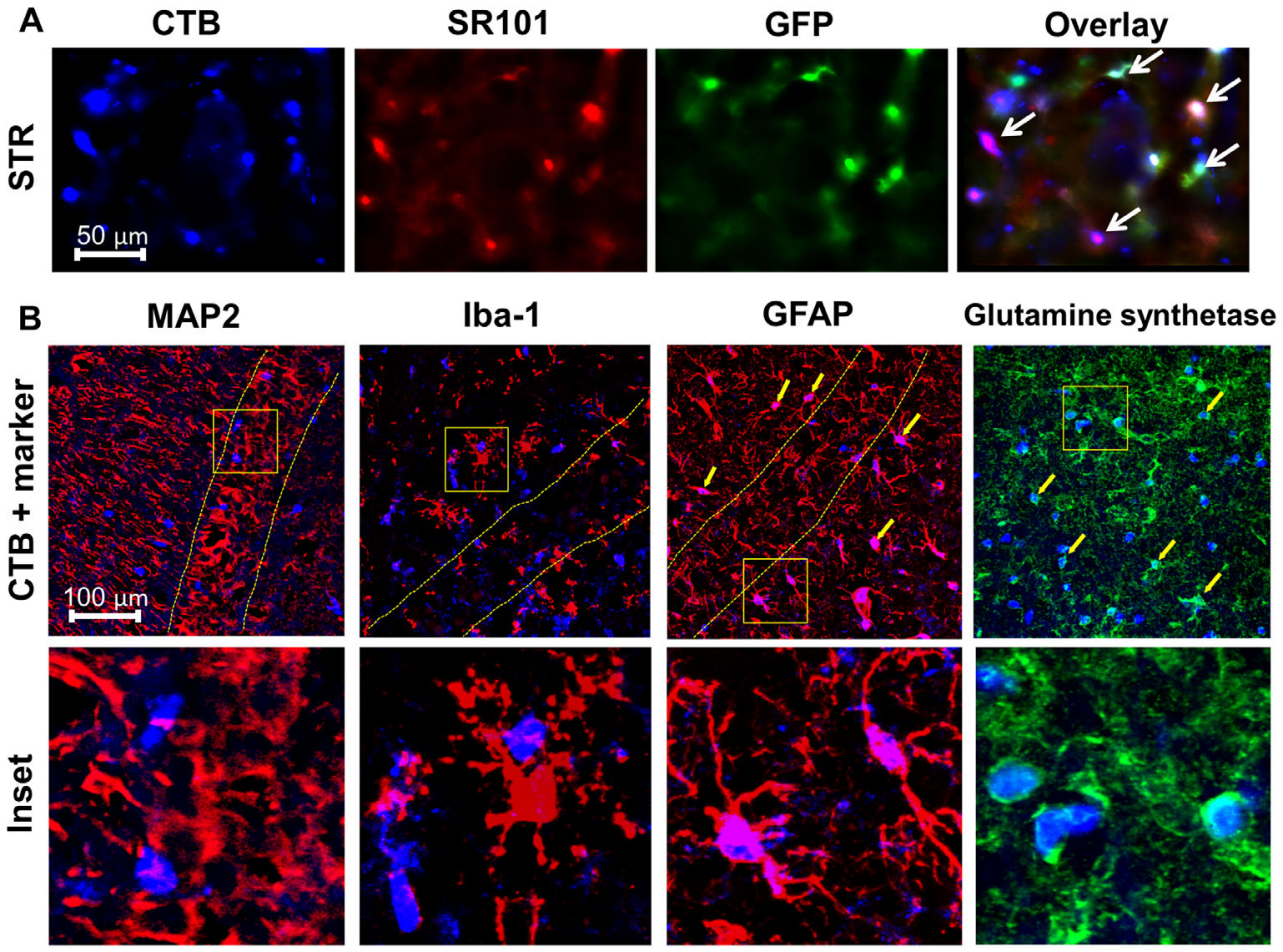
CellTracker Blue (CTB) co-localizes with traditional astrocyte markers. (A) Striatal (STR) brain slices from GFAP-GFP mice were stained with the astrocyte-selective dye sulforhodamine 101 (SR101) and CellTracker Blue (CTB). GFAP-GFP^+^ astrocytes (green) and SR101^+^ astrocytes (red) demonstrated a high degree of overlap with CTB (depicted by arrows in the overlay image). (B) CTB stained brain slices from the hippocampus (HPC) or S1C were fixed and sectioned, whereupon immunofluorescence staining was performed for the astrocyte-specific markers GFAP and glutamine synthetase as well as MAP2 and Iba-1 to identify neurons and microglia, respectively. The pyramidal layer of the HPC is delineated with dashed lines. Astrocytes demonstrating overlap with CTB and GFAP or glutamine synthetase are indicated by arrows.

Before we initiated our analysis of HC activity in CLN3^Δex7/8^ astrocytes, a method was needed to reliably identify cells in acute brain slices. Since the fluorescent dyes ethidium bromide (EtBr) and sulforhodamine 101 (SR101), which are commonly used for assessing HC activity and astrocyte identification, respectively [41], [48], have overlapping wavelengths, we examined a panel of fluorescent molecules that could be used for astrocyte detection with excitation/emission properties distinct from EtBr. This led us to develop a staining protocol using the fluorescent dye CTB (7-amino-4-chloromethylcoumarin, CMAC). CTB is a membrane-permeable thiol-reactive probe, which undergoes a glutathione *S*-transferase–mediated reaction intracellularly to produce a membrane-impermeable glutathione fluorescent adduct [49], [50]. Importantly, astrocytes are rich in glutathione [51] and can be clearly distinguished from neurons by CTB staining in most brain regions based on their size and morphological characteristics [49], [50]. To confirm astrocyte staining with CTB in acute brain slices, we utilized GFAP-GFP transgenic mice, where both CTB and the classical astrocyte-selective dye SR101 were found to overlap with GFAP-GFP^+^ astrocytes (Figure 1A). To further confirm CTB uptake in astrocytes, we performed immunofluorescence staining for the astrocyte-specific molecules GFAP and glutamine synthetase. CTB showed extensive overlap with both GFAP and glutamine synthetase in numerous brain regions, including the HPC and S1C (Figure 1B, and data not shown). Importantly, MAP2 and Iba-1 staining, to assess CTB uptake in neurons and microglia, respectively, revealed no co-localization with CTB in either population (Figure 1B), confirming that astrocytes are the primary cell type labeled by CTB in acute brain slices. Moreover, patch-clamp recordings of live CTB^+^ cells in acute brain slices confirmed that cells possessed characteristic astrocyte properties in several brain regions (data not shown). Based on this evidence, we conclude that the vast majority of CTB stained cells in live brain slices were astrocytes. Although it remains possible that other CNS cell types may internalize CTB, especially when higher dye concentrations are utilized, this was not a concern in our studies, since CTB levels were low (i.e. 2 µM). Because CTB displayed consistent cellular retention properties and photostability, and fluorescent emission was not promiscuous across other wavelengths, CTB was utilized in these studies to identify astrocytes when measuring HC activity in acute brain slices from CLN3^Δex7/8^ and WT mice.

### Region- and Age-dependent Accumulation of Lysosomal Ceroid Inclusions in CLN3^Δex7/8^ Mice

**Figure 2:**
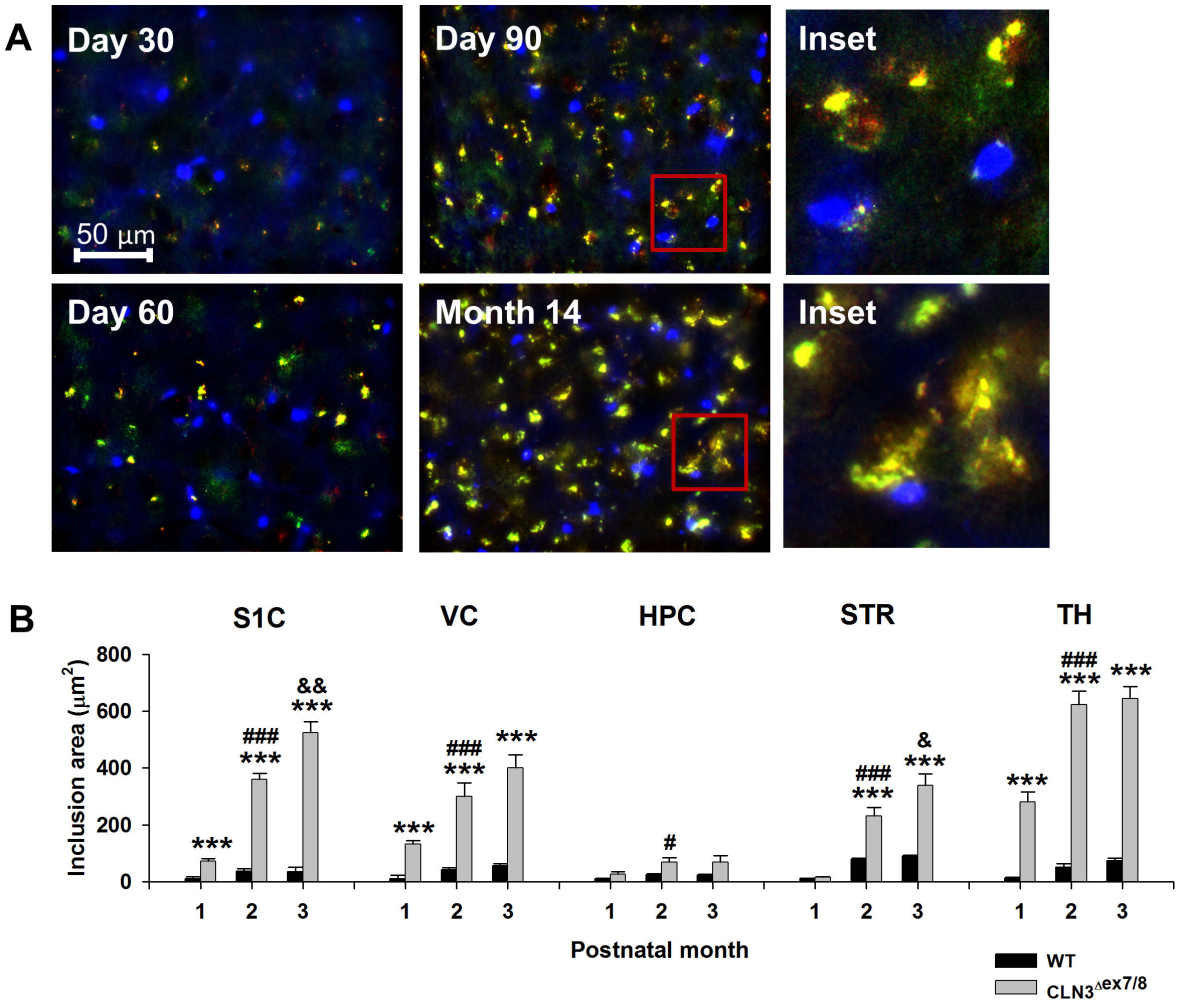
CLN3^Δex7/8^ mice display age- and region-dependent accumulation of lysosomal ceroid inclusions. (A) Examples of autofluorescent ceroid inclusions in acute brain slices from the visual cortex of CLN3^Δex7/8^ mice at day 30, 60, 90 and 14 months. Inclusions are visible in both the GFP (green) and rhodamine (red) filters (overlay = yellow-orange). CTB stained astrocyte soma (blue) displayed only a few intracellular inclusions (see insets). (B) Age-dependent accumulation of ceroid inclusions within the somatosensory cortex (S1C), visual cortex (VC), hippocampus (HPC), striatum (STR), and thalamus (TH) of wild type (WT) and CLN3^Δex7/8^ mice (5–8 animals per group). Significant differences between WT and CLN3^Δex7/8^ tissues are denoted by asterisks (***p<0.001), whereas changes between CLN3^Δex7/8^ tissues at postnatal days 30 and 60 within the same brain region are indicated by hatched signs (#, *p*<0.05; ###, *p*<0.001), and differences between CLN3^Δex7/8^ tissues at postnatal days 60 and 90 are indicated by ampersands (&&, *p*<0.01).

The CLN3^Δex7/8^ mouse model of JNCL displays increased accumulation of autofluorescent ceroid inclusions, which is evident at early postnatal ages [52]. To facilitate downstream comparisons in astrocyte HC/GJ activity with disease pathology, we first evaluated the extent of ceroid inclusions in CLN3^Δex7/8^ mice, focusing on brain regions where subsequent analysis of astrocyte HC activity was performed. In agreement with previous reports [52], neurons displayed the most prominent accumulation of inclusions, although smaller deposits of storage material were also observed in CTB stained astrocytes (Figure 2A). In general, inclusion formation progressively increased with age in CLN3^Δex7/8^ mice in all five brain regions examined (Figure 2B). Intracellular accumulation of lipofuscin is a natural process associated with aging and, as such, WT animals also displayed a low degree of inclusion material [14]. However, inclusions were significantly higher in CLN3^Δex7/8^ animals in the S1C, VC, and TH at all ages compared to WT mice (Figure 2B). Interestingly, the HPC had low inclusion burdens at all three time points examined, even for CLN3^Δex7/8^ animals. This may be explained by the fact that the stratum radiatum layer of the hippocampus, which was examined here, has few neurons. Notably, the most pronounced expansion of inclusions in CLN3^Δex7/8^ mice occurred between postnatal days 30 and 60 in all five brain regions examined, whereas increases were less pronounced between days 60 and 90 (Figure 2B). Based on this analysis, the severity of inclusion deposition in CLN3^Δex7/8^ mice was ranked from most to least affected, namely TH<S1C<VC<STR<HPC. This relationship may be influenced by the relative numbers of neuronal cell bodies present in each structure, since neurons readily accumulate storage material. No significant differences in ceroid deposition were observed between male and female CLN3^Δex7/8^ animals (data not shown).

### CLN3^Δex7/8^ Astrocytes Display Transient Region-dependent Increases in HC Activity that Decline with Advancing Age

**Figure 3:**
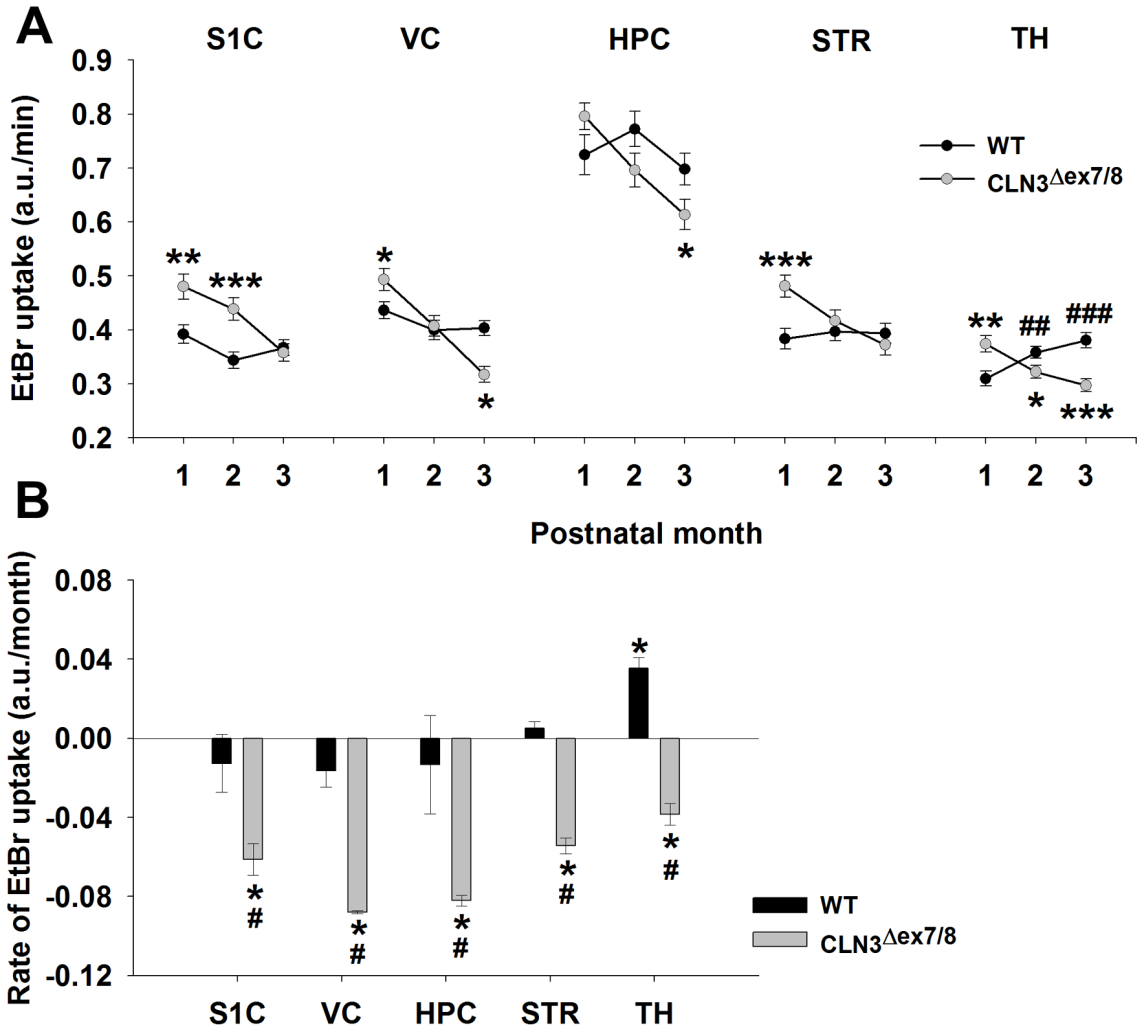
CLN3^Δex7/8^ astrocytes display transient region-dependent increases in hemichannel (HC) activity that decline with advancing age. Acute brain slices were prepared from wild type (WT) and CLN3^Δex7/8^ mice at postnatal days 30, 60 and 90, whereupon (A) EtBr uptake was measured in astrocytes in the somatosensory cortex (S1C), visual cortex (VC), hippocampus (HPC), striatum (STR), and thalamus (TH) as a quantitative measure of HC activity, which is expressed in arbitrary units (a.u.) per min. (B) Linear regression coefficients were determined for each brain region over time using the data shown in (A) and are presented as a.u. per month. Significant differences between WT and CLN3^Δex7/8^ astrocyte HC activity are indicated by asterisks (*p<0.05; **p<0.01; ***p<0.001), whereas changes within either WT or CLN3^Δex7/8^ astrocytes in the same brain region over time are denoted by hatched signs (#, *p*<0.05; ##, *p*<0.01; ###, *p*<0.001).

Astrocytes are recognized for their role in maintaining tissue homeostasis, energy metabolism, and cell-cell communication, in part, through GJ and HC function [25], [26]. Both astrocyte HC and GJ activity can be dramatically affected during pathological conditions and in some neurological diseases [41], [42], [43], which likely perturbs CNS homeostasis and the brain metabolome. Therefore, we examined HC and GJ activity of CLN3^Δex7/8^ and WT astrocytes in the same five brain regions with advancing age, to determine whether a link could be established between changes in astrocyte communication and ceroid accumulation. We elected to study younger animals with the eventual goal of identifying abnormalities that could be targeted to delay/prevent neuronal loss during later stages of JNCL. HC activity was significantly increased in CLN3^Δex7/8^ astrocytes at postnatal day 30 in four of the five brain regions examined, including the S1C, VC, STR, and TH (Figure 3A). However, increased HC opening in CLN3^Δex7/8^ mice was transient, where, in general, HC activity progressively decreased compared to WT animals at postnatal days 60 and 90 (Figure 3A). Interestingly, although HC activity steadily declined in CLN3^Δex7/8^ mice, it remained relatively stable in WT animals across postnatal days 30–90 (Figure 3B). The S1C of CLN3^Δex7/8^ mice were unique in that this region maintained open astrocyte HCs at postnatal day 60, whereas other brain areas displayed decreased activity. No differences in astrocyte HC activity were detected between male and female CLN3^Δex7/8^ mice (data not shown).

We next examined astrocyte GJC in the S1C and HPC during whole-cell patch clamp recordings. Only two brain regions could be examined due to the time-intensive nature of electrophysiology studies. No significant differences were observed between CLN3^Δex7/8^ and WT astrocytes in the S1C at any postnatal day examined (Figure S1). In contrast, GJC was significantly increased in CLN3^Δex7/8^ astrocytes in the HPC at postnatal day 90 compared to WT cells (40.2±2.6, n = 18 vs. 31.3±2.7, n = 15, respectively; *p*<0.05) (Figure S1). Collectively, these results are the first to demonstrate changes in astrocyte HC/GJ activity in the context of CLN3 mutation, which may have an impact on disease progression.

### CLN3 Mutation is Associated with the Decreased Expression of Molecules Involved in Glutamate Homeostasis

We next investigated whether the observed changes in CLN3^Δex7/8^ astrocyte HC activity coincided with alterations in the expression of key proteins implicated in CNS homeostasis. In particular, molecules important for glutamate regulation were examined given the reported role of glutamate excitotoxicity during JNCL [53], [54], [55]. We chose to utilize immunofluorescence staining and confocal microscopy to obtain maximal image resolution; however, we first needed to address the autofluorescent ceroid inclusions in CLN3^Δex7/8^ mice, which can interfere with the detection of target proteins by immunofluorescence staining and confound data interpretation. This issue was resolved by incubating tissue sections with Sudan black, which quenched the autofluorescence associated with CLN3^Δex7/8^ tissues (Figure S2). Sudan Black is known to interact with lipophilic inclusions several disorders, including Batten disease [56], [57] and is widely used for attenuating undesirable autofluorescence in numerous tissues [58]. This approach allowed us to evaluate the expression of key astrocyte molecules in CLN3^Δex7/8^ mice using immunofluorescence staining methods.

**Figure 4:**
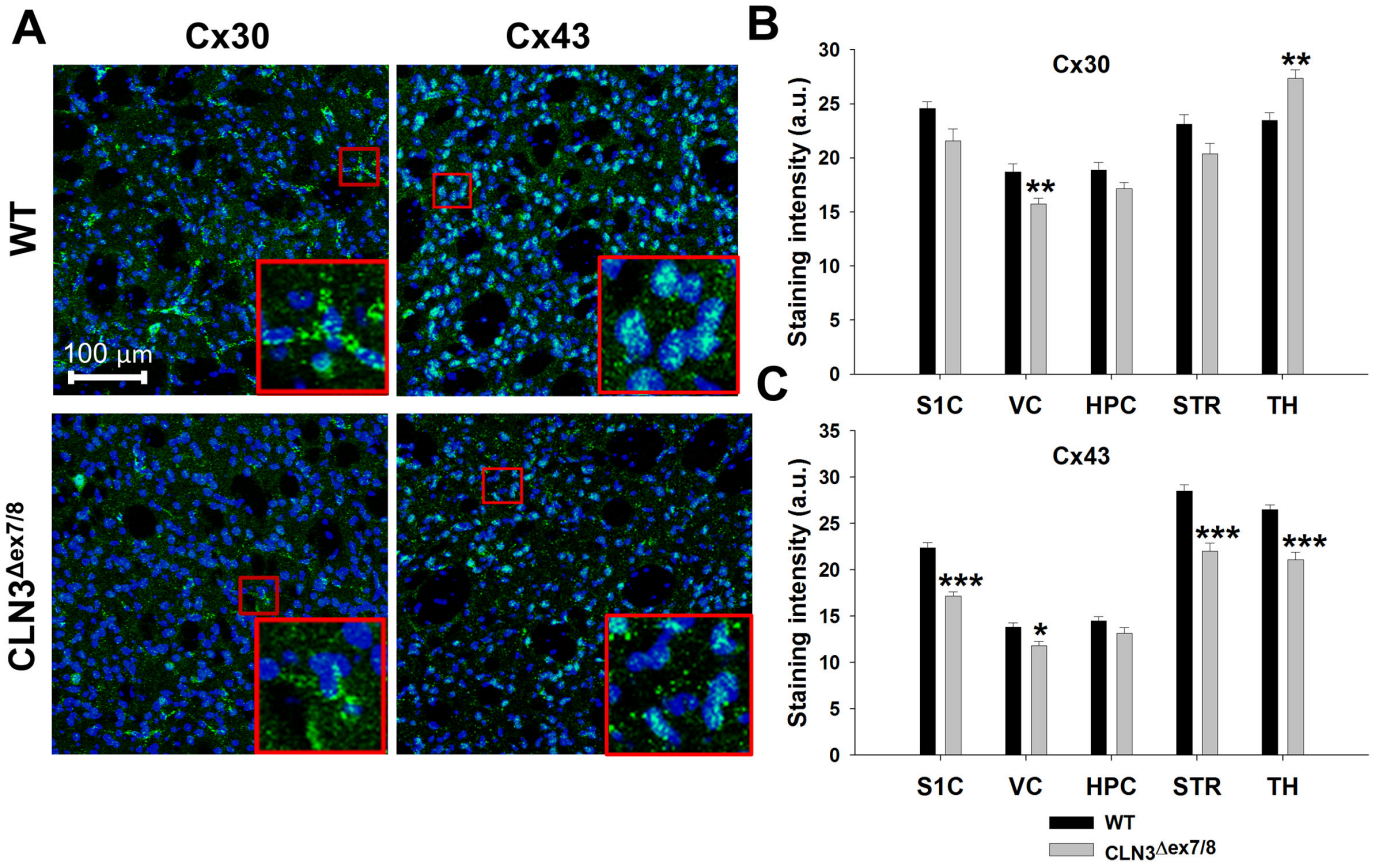
Connexin expression is significantly reduced in CLN3^Δex7/8^ astrocytes in a region-dependent manner. Immunofluorescence staining for Cx30 and Cx43 was performed on tissue sections from the somatosensory cortex (S1C), visual cortex (VC), hippocampus (HPC), striatum (STR), and thalamus (TH) of wild type (WT) and CLN3^Δex7/8^ mice at postnatal day 90. (A) Representative images depicting Cx30 and Cx43 expression in the STR with nuclei depicted by DAPI staining (blue; magnification, 20X; inset 50×50 µm zoom). (B) Cx30 and (C) Cx43 immunoreactivity was quantitated in each brain region (n = 3–4 mice per group; 6 images/region for each animal) with results reported in arbitrary units (a.u.). Significant differences in Cx30 and Cx43 staining between WT and CLN3^Δex7/8^ tissues are denoted by asterisks (*p<0.05; **p<0.01; ***p<0.001).

Cx43 is a main component of astrocyte GJs and HCs, although Cx30 and pannexin 1 have also been implicated in GJ and HC formation, respectively [25], [26]. We next quantitated Cx43 and Cx30 staining in the same five brain regions of CLN3^Δex7/8^ and WT mice where HC function was assessed, to determine whether protein expression coincided with the observed changes in astrocyte HC activity. Protein levels were only examined at postnatal day 90 since this was the latest interval assessed in this study and therefore, would represent the most severe pathology compared to earlier time points. Interestingly, Cx43 expression was significantly lower in the S1C, VC, STR, and TH of CLN3^Δex7/8^ mice compared to WT animals (Figure 4A and C) but not in the HPC, where GJC was significantly increased in CLN3^Δex7/8^ mice at the same age (i.e. postnatal day 90; Figure S1). In contrast, Cx30 was more variable, with expression significantly decreased and increased in the VC and TH, respectively, whereas other brain regions displayed similar levels (Figure 4A and B).

**Figure 5:**
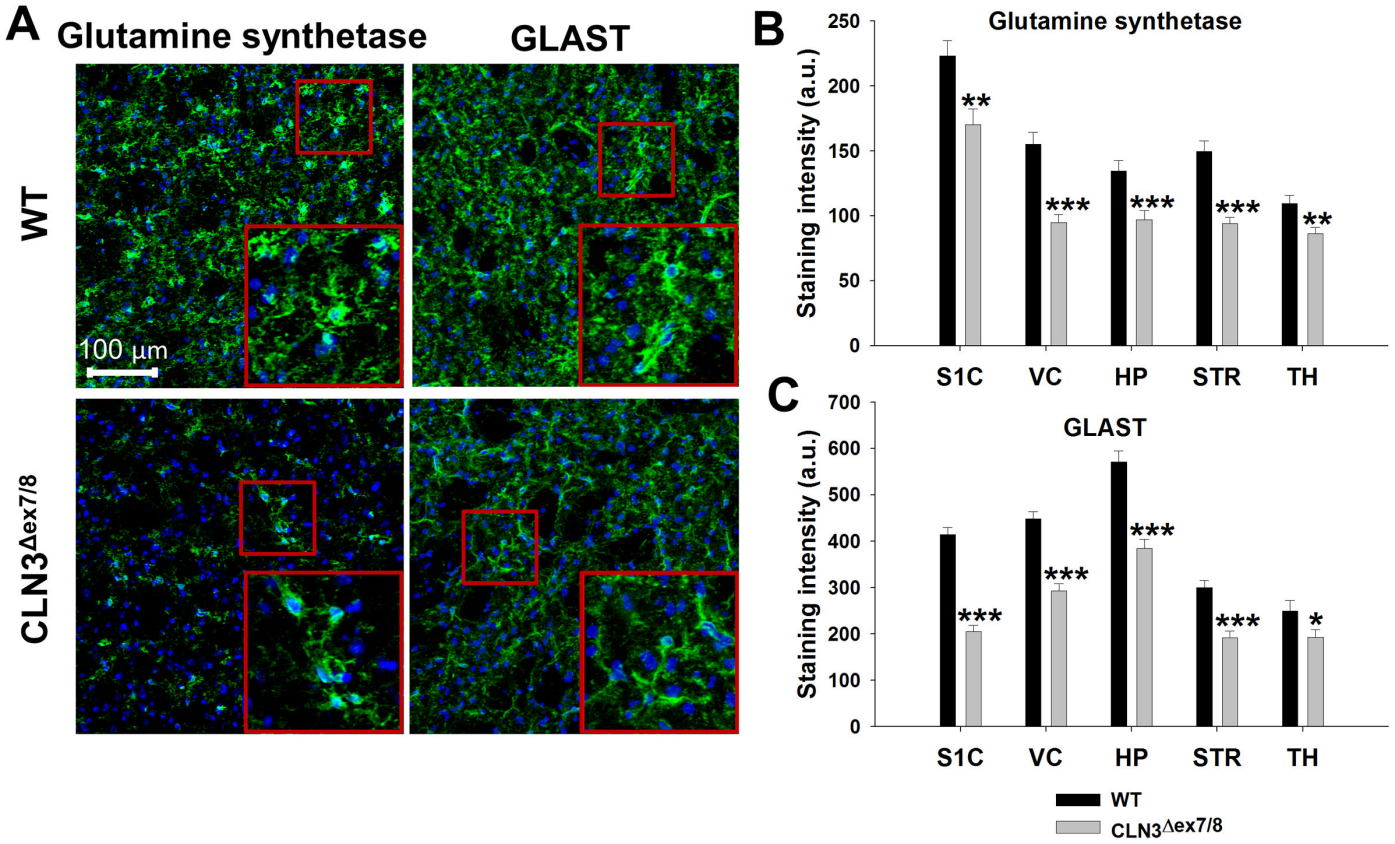
CLN3^Δex7/8^ astrocytes display significant reductions in molecules critical for glutamate homeostasis. Immunofluorescence staining for glutamine synthetase and GLAST was performed on tissue sections from the somatosensory cortex (S1C), visual cortex (VC), hippocampus (HPC), striatum (STR), and thalamus (TH) of wild type (WT) and CLN3^Δex7/8^ mice at postnatal day 90. (A) Representative images depicting glutamine synthetase and GLAST expression in the STR with nuclei depicted by DAPI staining (blue; magnification, 20X; inset 50×50 µm zoom). (B) Glutamine synthetase and (C) GLAST immunoreactivity was quantitated in each brain region (n = 3–4 mice per group; 6 images/region for each animal) with results reported in arbitrary units (a.u.). Significant differences in glutamine synthetase and GLAST staining between WT and CLN3^Δex7/8^ tissues are denoted by asterisks (*p<0.05; **p<0.01; ***p<0.001).

**Figure 6:**
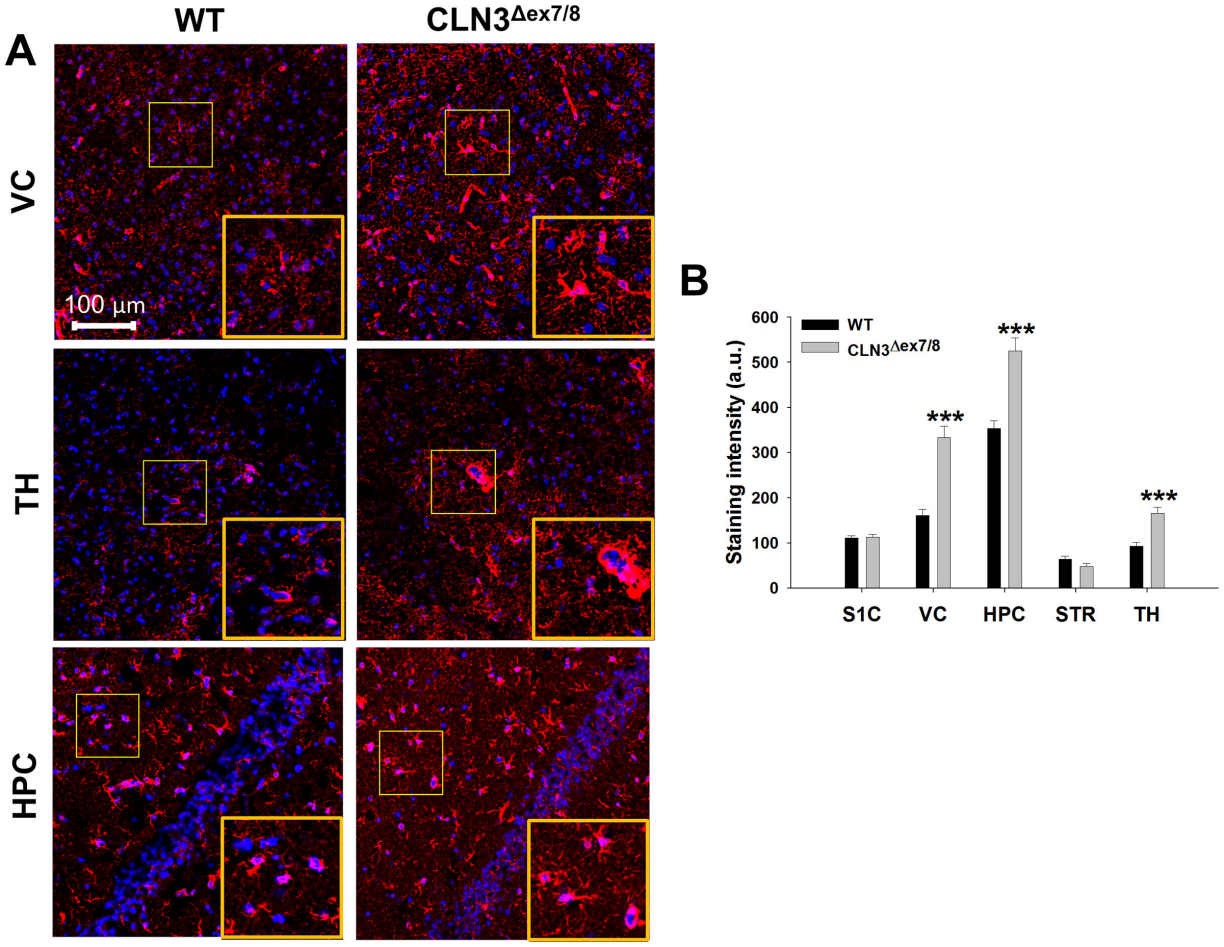
GFAP expression is significantly increased in select brain regions of CLN3^Δex7/8^ mice. Immunofluorescence staining for GFAP was performed on tissue sections from the somatosensory cortex (S1C), visual cortex (VC), hippocampus (HPC), striatum (STR), and thalamus (TH) of wild type (WT) and CLN3^Δex7/8^ mice at postnatal day 90. (A) Representative images depicting GFAP expression in the VC, TH, and HPC with nuclei depicted by DAPI staining (blue; magnification, 20X; inset 50×50 µm zoom). (B) GFAP immunoreactivity was quantitated in each brain region (n = 3–4 mice per group; 6 images/region for each animal) with results reported in arbitrary units (a.u.). Significant differences in GFAP staining between WT and CLN3^Δex7/8^ tissues are denoted by asterisks (***p<0.001).

Glutamine synthetase plays an important role in glutamate metabolism, converting glutamate into glutamine, the latter of which is utilized as a substrate for glutamate production in neurons [19]. In addition, glutamate levels are elevated in the brains of JNCL patients and CLN3 mutant mice [59], [60], [61], and since astrocytes exclusively express glutamate synthetase in the CNS, altered enzyme levels could represent a key mechanism responsible for glutamate accumulation in the JNCL brain. Likewise, the glutamate transporter GLAST is a key pathway for glutamate uptake in astrocytes and could also be implicated in progressive disease pathology. Similar to Cx43, both glutamate synthetase and GLAST expression were significantly reduced in CLN3^Δex7/8^ mice compared to WT animals at postnatal day 90 in all brain regions examined (Figure 5). These decreases in Cx43, GLAST, and glutamine synthetase in CLN3^Δex7/8^ animals did not result from astrocyte loss, since GFAP expression was significantly elevated in the VC, HPC, and TH (Figure 6) in agreement with previous reports from other laboratories in CLN3 mutant mice [8], [20]. This is a key observation, since it reveals the attrition of molecules that regulate glutamate homeostasis in CLN3^Δex7/8^ animals, which likely triggers astrocyte activation in an attempt to rectify this decline. Western blot analysis also revealed significant reductions in Cx43 and glutamine synthetase expression in the S1C, VC, and HPC (Figures S3 and S4); however, some differences were noted in other brain regions compared to confocal microscopy findings. With regard to Cx43, this may be explained by the contributions of other Cx43-positive cell types in brain extracts, including microglia, endothelial cells, ependymal cells, and pericytes [62], [63], [64], [65], [66], [67] that results in less quantitative precision in Western blots compared to confocal microscopy, although undeniably astrocytes are the major source of Cx43 in the normal CNS. Western blots revealed few differences in Cx30 expression (Figure S5) in agreement with the modest changes detected by immunofluorescence staining. In contrast, Western blotting did not reveal dramatic differences in GLAST expression (Figure S6), which again may be explained by reduced sensitivity compared to immunofluorescence staining. In addition, Western blots are semi-quantitative at best, whereas confocal microscopy is more sensitive at detecting subtle differences in protein levels and immunostaining has been a hallmark method for visualizing differences in glial molecules in JNCL [8], [20]. Finally, confocal imaging provides superior precision to reproducibly locate specific brain substructures between different animals (i.e. layers VI-IV of visual cortex) to improve the accuracy of signal quantitation.

### CLN3^Δex7/8^ Astrocytes Display Altered Membrane Properties

**Figure 7:**
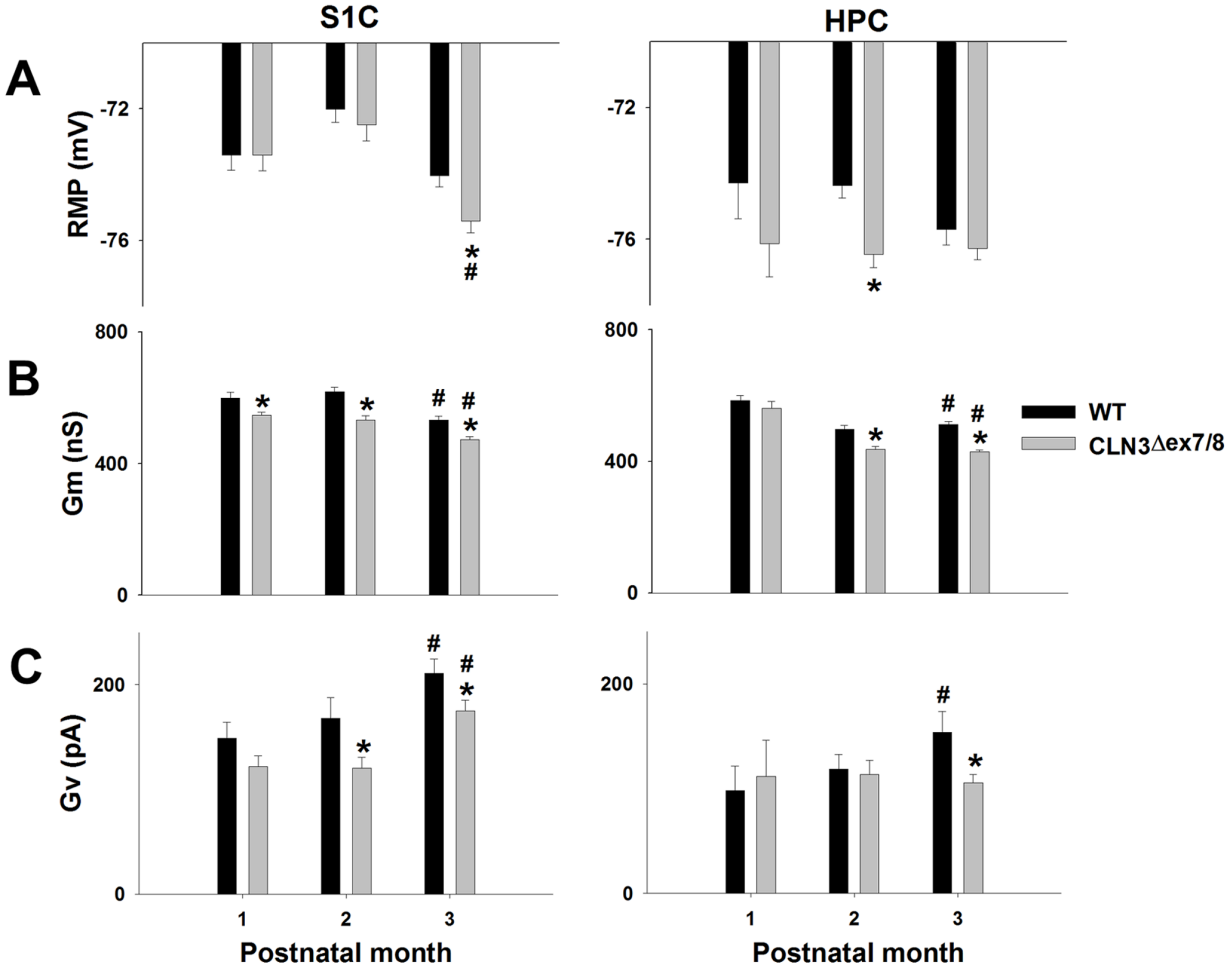
CLN3^Δex7/8^ astrocytes display reductions in membrane potential and conductance. Acute brain slices were prepared from wild type (WT) and CLN3^Δex7/8^ mice at postnatal days 30, 60 and 90, whereupon astrocyte resting membrane potential (RMP; A), resting membrane conductance (Gm; B), and voltage-dependent membrane conductance (Gv; C) were measured in the somatosensory cortex (S1C) and hippocampus (HPC) using whole-cell patch clamp recordings. Significant differences between WT and CLN3^Δex7/8^ astrocytes are denoted by asterisks (*p<0.05), whereas changes between values at postnatal days 30 versus 60 or 90 are indicated by hatched signs (#, *p*<0.05). Refer to Tables S1 and S2 for statistical values.

Electrophysiological recordings of astrocytes in acute brain slices of CLN3^Δex7/8^ and WT mice were made to determine whether any intrinsic defects were evident that could be linked to the observed changes in HC activity or disease progression/severity. No studies to date have evaluated the effects of CLN3 mutation on intrinsic astrocyte properties and our analysis of brain slices is superior to cultured astrocytes where complex interactions with other cell types are lost. In general, CLN3^Δex7/8^ astrocytes were more hyperpolarized compared to WT cells, as revealed by significant changes in RMP in both the S1C (−74.32±0.30, n = 228 vs. −73.31±0.25 mV, n = 216, respectively; *p*<0.05) and HPC (−76.59±0.24, n = 170 vs. −75.73±0.30 mV, n = 138, respectively; *p*<0.05) (Figure 7A). Extrapolation of Gm values revealed consistent reductions in Gm for CLN3^Δex7/8^ astrocytes compared to WT cells both in the S1C and HPC during all three postnatal periods examined, with the exception of day 30 in the latter (Figure 7B and Tables S1 and S2).

The most dramatic distinction between CLN3^Δex7/8^ and WT astrocytes was in voltage-dependent conductance (Gv). Specifically, CLN3^Δex7/8^ astrocytes revealed significantly decreased Gv compared to WT cells in the S1C (152.3±7.03, n = 292 vs. 187.7±9.56 pA, n = 293, respectively; *p*<0.001) (Figure 7C). Similar decreases in Gv occurred in CLN3^Δex7/8^ astrocytes in the HPC, but were only evident at postnatal day 90 (Figure 7C). In general, decreased Gv in CLN3^Δex7/8^ astrocytes correlates with the observed inhibition of astrocyte HC activity with advancing postnatal age.

### The CBX Derivative INI-0602 Attenuates Astrocyte HC Activity in Acute Brain Slices

**Figure 8:**
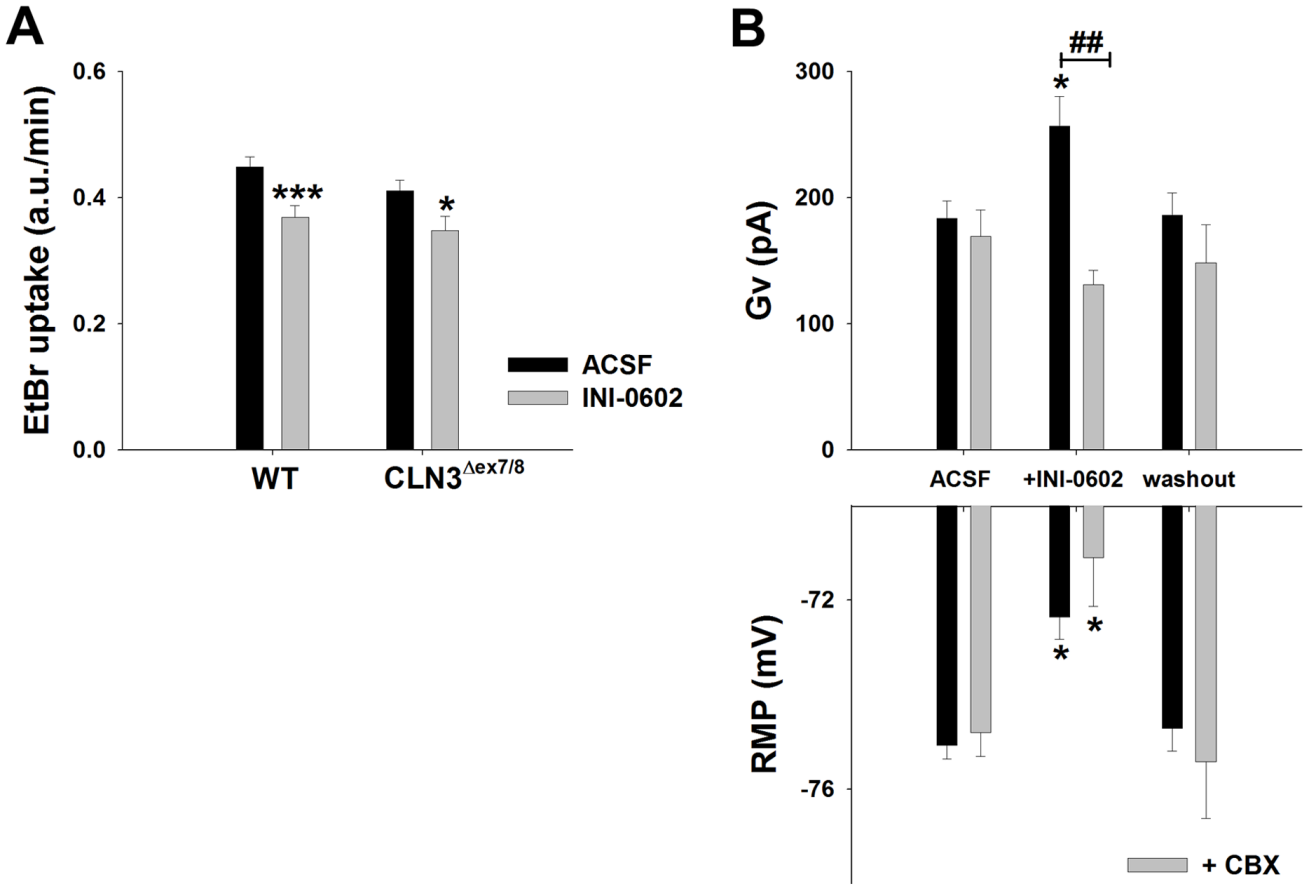
Bath application of INI-0602 inhibits HC activity and alters astrocyte electrophysiological properties. Acute brain slices were prepared from wild type (WT) and CLN3^Δex7/8^ mice at postnatal day 90, whereupon measurements were made in the somatosensory cortex. (A) Direct application of INI-0602 (100 µM) in the bath solution inhibited HC activity of both WT and CLN3^Δex7/8^ astrocytes as measured by the rate of EtBr uptake (presented in arbitrary units (a.u.) per minute). (B) INI-0602 bath application increases voltage-dependent membrane conductance (Gv) of WT astrocytes and evokes membrane depolarization (RMP). Simultaneous bath application of INI-0602 and its parental compound carbenoxolone (CBX; 50 µM) prevented the Gv increase but not RMP depolarization. In (A), significant differences between astrocytes in ACSF alone versus ACSF+INI-0602 are denoted by asterisks (*p<0.05; ***p<0.001), whereas in (B), significant differences between WT astrocytes bathed in ASCF only versus INI-0602 or INI-0602+ CBX are indicated by asterisks (*p<0.05) and alterations between WT astrocytes bathed in INI-0602 versus INI-0602+ CBX are denoted by hatched signs (##, *p*<0.01).

A recent report described the generation of a novel blood-brain barrier permeable HC inhibitor, INI-0602, which is a derivative of the well-known HC/GJ inhibitor carbenoxolone (CBX) [68]. Treatment with INI-0602 in mouse models of amyotrophic lateral sclerosis and Alzheimer’s disease led to significant improvements in motor activity, survival, and cognitive function [68]. Since astrocyte HC activation was increased in CLN3^Δex7/8^ mice at postnatal day 30, we first tested the ability of INI-0602 to inhibit astrocyte HCs in acute brain slices *in vitro* as proof-of-principle. INI-0602 significantly inhibited EtBr uptake in the S1C of both WT and CLN3^Δex7/8^ slices at postnatal day 90 compared with control tissues incubated in ACSF alone (Figure 8A). Importantly, HC activity was similar in CLN3^Δex7/8^ and WT slices bathed in ACSF alone, confirming our earlier findings in the S1C at postnatal day 90 (Figure 3A). INI-0602 also induced changes in astrocyte electrophysiological parameters (Figure 8B) and only WT slices were examined in these experiments to demonstrate drug action on normal cells. Brief INI-0602 application (i.e. 5–10 min) attenuated Gm values in WT astrocytes (365.86±5.96, n = 417 vs. 308.73±4.21 nS, n = 417 vs. 331.48±4.37, n = 280 for ACSF, INI-0602, and drug washout, respectively; *p*<0.05) followed by a depolarizing inward current (−164.2±28.6 pA; range −30 to −600 pA). Gv was significantly increased during INI-application compared to ASCF alone (256.6±23.4 vs. 183.3±14.0 pA, respectively; *p*<0.05). The parental compound CBX prevented the increase in Gv but not membrane depolarization induced by INI-0602 in WT astrocytes (Figure 8B). Since the effects of INI-0602 on intrinsic astrocyte properties could be washed out, this suggests that INI-0602 exerts rapid and reversible changes in astrocyte membrane properties.

### The HC Inhibitor INI-0602 Reduces Lysosomal Ceroid Inclusions, Enhances GJC, and Restores Astrocyte RMP in CLN3^Δex7/8^ Mice

**Figure 9:**
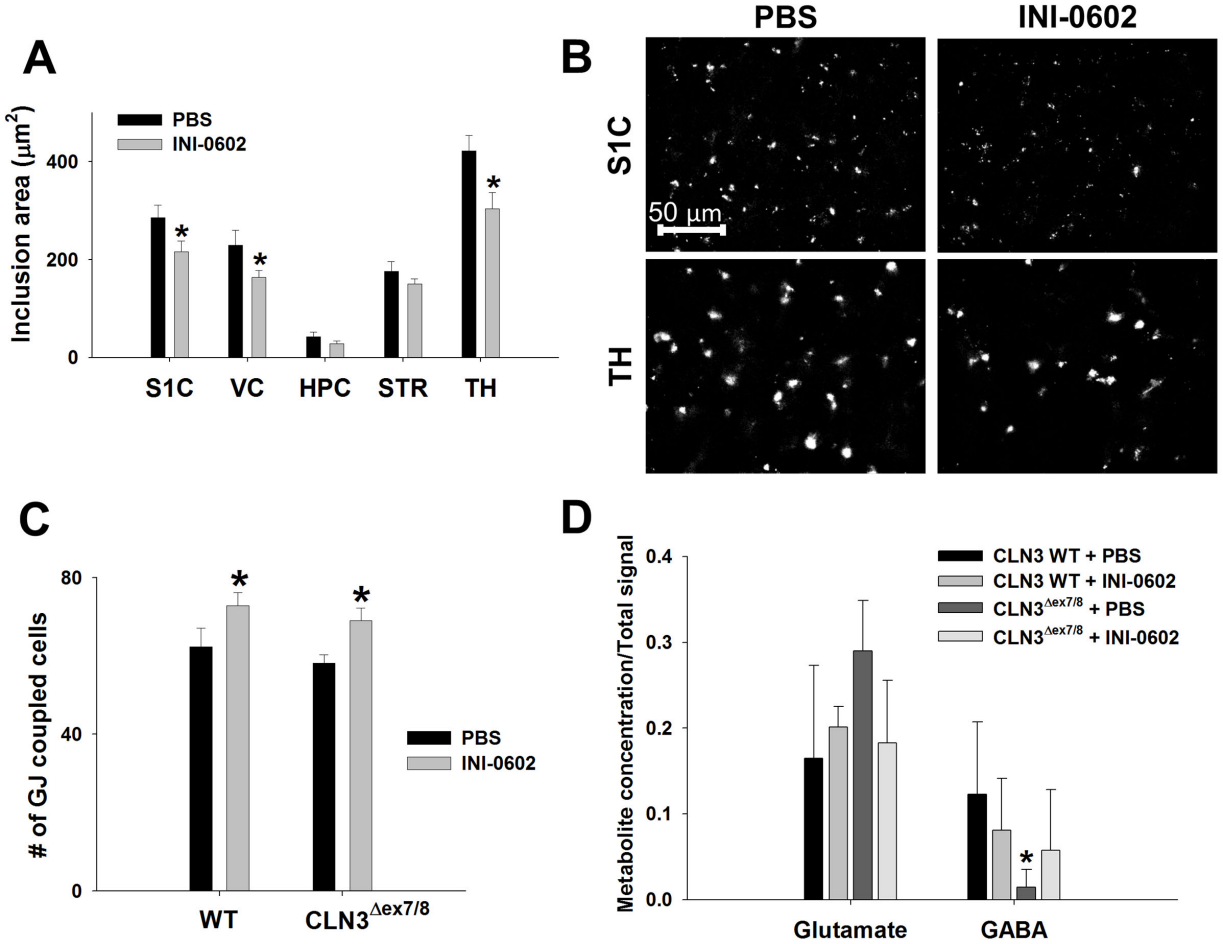
*In vivo* administration of the HC inhibitor INI-0602 reduces ceroid inclusions in CLN3^Δex7/8^ mice and enhances gap junction communication (GJC). CLN3^Δex7/8^ and wild type (WT) mice were treated with INI-0602 (10 mg/kg) or PBS from postnatal day 30 to 60, whereupon measurements were conducted (n = 4–8 mice/group). (A) The area of lysosomal ceroid inclusions in CLN3^Δex7/8^ mice receiving INI-0602 or PBS was quantitated in the somatosensory cortex (S1C), visual cortex (VC), hippocampus (HPC), striatum (STR), and thalamus (TH). (B) Representative images of ceroid inclusions in the S1C or TH of CLN3^Δex7/8^ mice treated with PBS or INI-0602. (C) The number of gap junction (GJ) coupled astrocytes was evaluated in the S1C by monitoring passage of the GJ permeable dye AlexaFluor 350 (n = 21 and 24 cells for PBS- vs. INI-0602-treated WT mice, respectively; n = 36 and 39 cells for PBS- vs. INI-0602-treated CLN3^Δex7/8^ mice, respectively). (D) Glutamate and GABA concentrations in the HPC as measured by magnetic resonance (MR) spectroscopy (n = 4–5 mice/group). Significant differences are indicated by asterisks (*p<0.05).

Since astrocyte HC activity was increased in several brain regions of CLN3^Δex7/8^ mice at postnatal day 30 and could be blocked by INI-0602 in acute brain slices *in vitro*, we next treated CLN3^Δex7/8^ and WT mice with INI-0602 for a one month period, spanning from postnatal day 30 to 60, to examine its impact on disease parameters. Strikingly, a one month treatment period of CLN3^Δex7/8^ mice with INI-0602 significantly decreased lysosomal ceroid inclusions in three brain regions with the most pronounced accumulation, namely the S1C, VC, and TH compared to CLN3^Δex7/8^ mice receiving PBS (Figure 9A and B).

To determine whether *in vivo* INI-0602 administration had any effects on astrocyte HC/GJ activity, we examined both parameters in acute brain slices recovered from CLN3^Δex7/8^ and WT mice treated with INI-0602 for a one month period. Astrocyte GJC was significantly increased in both CLN3^Δex7/8^ (INI-0602∶71.9±3.5, n = 32 vs. PBS: 57.9±3.0 cells, n = 31, *p*<0.01) and WT mice (INI-0602∶73.9±3.6, n = 22 vs. PBS: 59.7±5.2 cells, n = 22, *p*<0.05) compared to PBS-treated controls (Figure 9C). Because INI-0602 enhanced GJC, we expected HC activity to be inhibited based on the typical reciprocal relationship reported between these modes of communication in several CNS models [41], [42]. Although astrocyte HC opening was significantly elevated in the S1C, VC, HPC, and STR of CLN3^Δex7/8^ mice compared to WT animals, a one month dosing interval with INI-0602 did not reduce HC activity to WT levels (Figure S7). Of note, in these experiments, astrocyte HC activity was significantly increased in CLN3^Δex7/8^ mice at postnatal day 60 in all brain regions examined except the TH, which was not observed in earlier studies (Figure 3A). This may reflect a stress response in CLN3^Δex7/8^ animals precipitated by repeated i.p. injections during the one month dosing period, since elevated HC activity was also observed in CLN3^Δex7/8^ mice receiving PBS. Collectively, these findings suggest that INI-0602 may exert its beneficial effects by enhancing homeostatic GJC by an unknown mechanism. One possibility is that INI-602 provokes transient astrocyte activation, as revealed by its ability to induce slight membrane depolarization (Figure 8B), which may overcome the progressive decline in astrocyte function suggested by the decreases in glutamine synthetase, GLAST, and Cx43 expression in CLN3^Δex7/8^ mice.

Magnetic resonance (MR) spectroscopy is a non-invasive method used to obtain quantitative metabolic information from living animals in serial longitudinal studies [69], [70]. MR spectroscopy-visible metabolites include glutamate, glutamine, N-acetyl aspartate (NAA), choline, creatine, and myo-inositol [71]; however, recent advances in curve fitting methodology allow for the detection of low-level metabolites, including GABA, glucose, glycine, alanine, aspartic acid, and taurine. A prior report using single-voxel MR spectroscopy revealed elevated glutamate concomitant with reduced GABA during JNCL [60]. We confirmed these observations in the HPC of CLN3^Δex7/8^ mice at postnatal day 60, as revealed by a trend towards elevated glutamate coincident with significantly reduced GABA levels compared to WT animals, although the former did not reach statistical significance (Figure 9D). Since INI-0602 significantly increased astrocyte GJC, we examined whether this would translate into improvements in the brain metabolome of CLN3^Δex7/8^ mice. In general, INI-0602 trended towards restoring glutamate and GABA levels in CLN3^Δex7/8^ animals to levels approaching that of WT mice, but again these differences were not statistically significant (Figure 9D). No other changes in the CNS metabolome were observed between CLN3^Δex7/8^ and WT animals at this early postnatal interval (data not shown).

**Figure 10:**
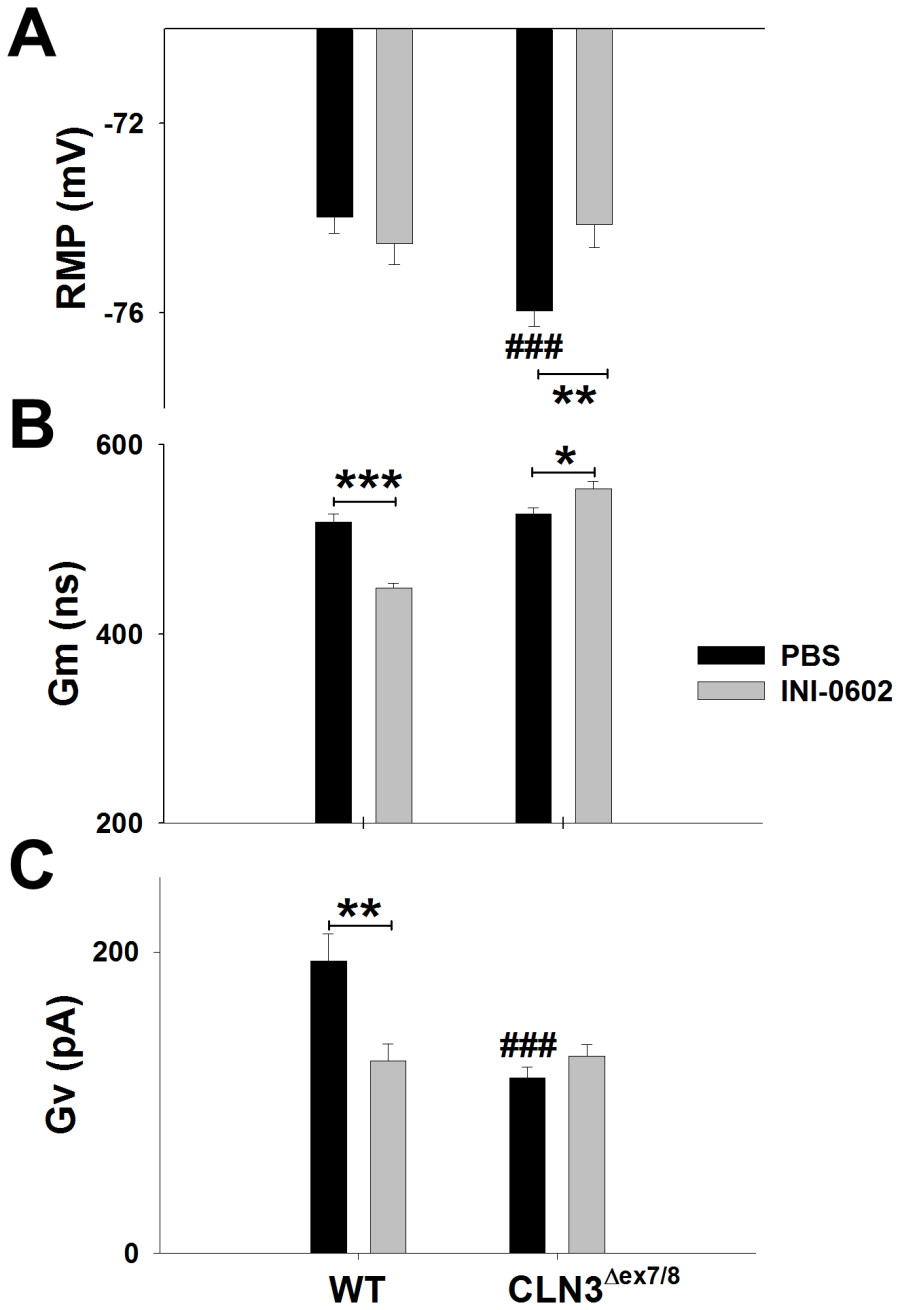
*In vivo* administration of the HC inhibitor INI-0602 alters astrocyte electrophysiological properties. CLN3^Δex7/8^ and wild type (WT) mice (n = 6–9/group) were treated with INI-0602 (10 mg/kg) or PBS from postnatal day 30 to 60, whereupon acute brain slices were prepared to quantitate resting membrane potential (RMP; A), resting membrane conductance (Gm; B), and voltage-dependent membrane conductance (Gv; C) in astrocytes from the somatosensory cortex using whole-cell patch clamp recordings. Significant differences between WT and CLN3^Δex7/8^ astrocytes are denoted by hatched signs (###, *p*<0.001), whereas changes between WT or CLN3^Δex7/8^ mice treated with INI-0602 or PBS are indicated by asterisks (*p<0.05; **p<0.01; ***p<0.001).

With regard to astrocyte electrophysiological properties, RMP was slightly hyperpolarized in astrocytes from CLN3^Δex7/8^ mice receiving PBS vehicle and returned to baseline values with INI-0602 treatment (Figure 10A). In addition, INI-0602 significantly increased astrocyte Gm in CLN3^Δex7/8^ mice, whereas Gv values were not affected. However, in comparison, the effects of INI-0602 on astrocyte Gm and Gv were more dramatic in WT animals (Figure 10B and C). Specifically, INI-0602 treatment significantly decreased Gm in WT animals compared to PBS-treated mice (517.8±9.1, n = 477 vs. 448.4±5.0 nS, n = 336, *p*<0.001; Figure 10B) as well as Gv (194.1±17.7, n = 92 vs. 127.8±11.0, n = 84 pA, *p*<0.01; (Figure 10C).

Although prior studies examining long-term dosing of mice with INI-0602 did not reveal any evidence of toxicity [68], blood chemistry profiles were performed to assess the safety profile of INI-0602 in the context of CLN3 mutation. INI-0602 did not alter basic blood parameters in either WT or CLN3^Δex7/8^ mice compared to their corresponding PBS controls (Table S3). However, CLN3^Δex7/8^ mice displayed reduced Na^+^ and Ca^2+^ compared to WT animals regardless of INI-0602 treatment. No significant weight changes were observed for any of the groups over the one month treatment period (data not shown). Collectively, these results demonstrate that INI-0602 exerts beneficial effects in the JNCL mouse model by reducing lysosomal ceroid inclusions, increasing astrocyte GJC, and normalizing astrocyte RMP without any untoward side effects. The mechanism of action for these findings remains to be fully elucidated; however, the increase in astrocyte GJC observed after a one month treatment period with INI-0602 is an attractive possibility.

## Discussion

Reactive astrocytes are a hallmark of JNCL in both the human brain and associated mouse models [8], [20], yet little information is available regarding the biological impact of astrocytes on disease pathogenesis. Based on the central role of astrocyte GJC/HC activity in controlling pH and ion balance, vascular control, neuronal activity at the tripartite synapse via glutamate regulation, and metabolic balance, it was envisioned that altered astrocyte function in the context of CLN3 mutation could influence JNCL pathology. CLN3^Δex7/8^ mice displayed increased HC activity at postnatal day 30 in many of the thalamocortical structures where neurons are destined to die at advanced age (i.e. ∼ 6 mo. onward), including the TH, STR, and cortical regions [8], [72]. Increased HC activity can lead to the dissipation of CNS homeostatic gradients due to the bidirectional communication between the intracellular and extracellular milieus [73]. One such effect emanating from HC opening is glutamate release and although it is tempting to speculate that astrocyte HCs are responsible, in part, for the exaggerated glutamate levels in JNCL reported by other groups [53], [59], [61], the regulatory mechanisms involved are likely much more complex, since astrocyte HC opening was only transient in CLN3^Δex7/8^ animals. With regard to the brain metabolome, our MR spectroscopy findings revealed significantly decreased GABA in CLN3^Δex7/8^ mice. Although a prior report described elevated glutamate and reduced GABA in CLN3 knockout mice, this analysis was performed using postmortem samples [61]. Importantly, our MR spectroscopy data were collected from living animals, which is more reflective of changes in real time. N-acetyl aspartate (NAA) is a widely used as a surrogate to reflect neuron viability, since it is a neuron-selective metabolite that can be resolved by MR spectroscopy [74]. NAA levels were identical between WT and CLN3^Δex7/8^ mice (data not shown), revealing little evidence of early neuronal pathology in the HPC of CLN3^Δex7/8^ animals. This was not unexpected because neuronal loss has not been reported to occur until months 6–8 in CLN3^Δex7/8^ mice [8], [20] and our MR spectroscopy analysis was performed on 2 month-old animals. It is possible that alterations in the brain metabolome are more pronounced in regions that were not examined here; however, the scan time required to obtain high resolution MR data in living mice for accurate spectroscopy is significant (i.e. ∼ 1.5–2 h/mouse), which limited our analysis of numerous brain regions in this study. It is highly likely that more robust changes in the brain metabolome of CLN3^Δex7/8^ mice would be evident at advanced ages; however, we elected to examine animals relatively early in the disease process, since our objective was to intervene with a therapeutic approach during acute disease to delay eventual neuronal loss.

**Figure 11:**
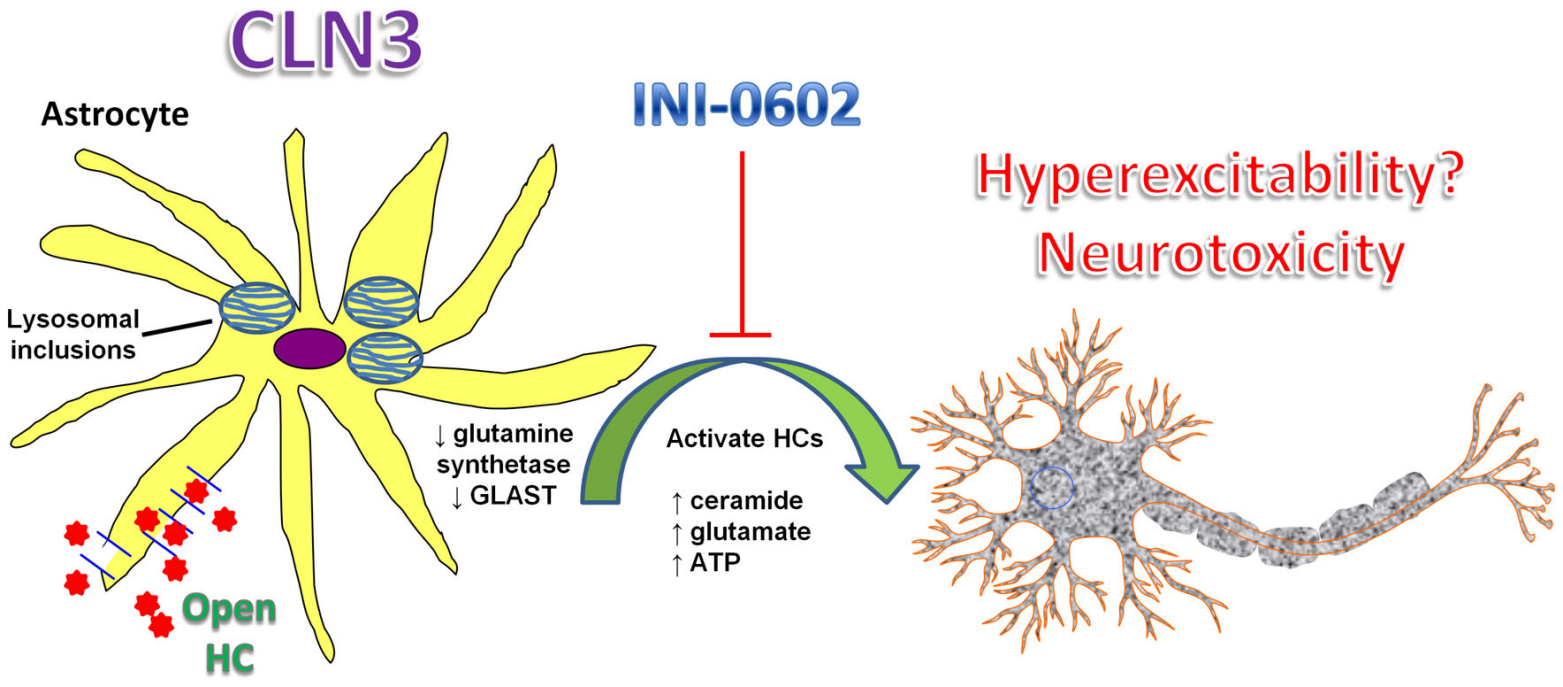
Proposed relationship between astrocyte HC activity, brain metabolites, and neuronal loss in JNCL. Astrocyte HCs are transiently opened in numerous brain regions in the context of CLN3 mutation, which likely distorts the brain metabolome. This is supported by the ability of the HC inhibitor, INI-0602, to reduce lysosomal ceroid inclusions and enhance gap junction communication that is associated with the maintenance of homeostatic gradients in the CNS milieu. Previous studies have reported increases in ceramide, ATP, and glutamate in JNCL, the latter of which can be linked to HC action as well as the global decreases in glutamine synthetase and GLAST expression in CLN3^Δex7/8^ mice. Collectively, these changes are suggestive of progressive loss of normal astrocyte homeostatic functions, which may contribute to neuronal loss in JNCL.

An intriguing finding in the current report was that astrocyte HC opening was only transiently increased in CLN3^Δex7/8^ mice at postnatal day 30. Beyond this interval, HC activity was significantly decreased in CLN3^Δex7/8^ animals compared to WT mice, which coincided with reduced membrane conductance and slight membrane hyperpolarization of CLN3^Δex7/8^ astrocytes both in the S1C and HPC (Figure 7). The reason(s) responsible for the progressive decline in CLN3^Δex7/8^ astrocyte activity are not clear; however, our data showing reduced glutamine synthetase and GLAST expression at postnatal day 90 are suggestive of progressive astrocyte dysfunction (Figure 11). These findings were bolstered by the observation that GFAP levels were significantly elevated in CLN3^Δex7/8^ mice, demonstrating selectivity in targeted molecules. Additional investigations into this possibility are necessary; nevertheless, the central role that astrocytes play in CNS maintenance and neuronal survival suggest this is a mechanism worth pursuing. The decrease in glutamine synthetase and GLAST levels in the CLN3^Δex7/8^ brain reported here is intriguing, since glutamate excitotoxicity represents an important mechanism of neuronal loss in JNCL [53], [55]. Reduced glutamine synthetase and GLAST expression may also originate from the protein trafficking defects that have been described in JNCL [75], [76], [77].

Based on the finding that astrocyte HC activity was significantly elevated at postnatal day 30 in CLN3^Δex7/8^ mice, coupled with published reports suggesting that HC opening can be linked with pathology, we treated CLN3^Δex7/8^ mice with the HC inhibitor INI-0602. The rationale for delayed administration at postnatal day 30 was to more closely model the age when a diagnosis of JNCL is made in children. Treatment of CLN3^Δex7/8^ animals with INI-0602 for a 30 day period significantly decreased lysosomal ceroid inclusions in several brain regions. In addition, the “passive” component of membrane conductance in CLN3^Δex7/8^ astrocytes (Gm) was also affected by INI-0602 treatment; however, its “reactive” voltage-dependent component (Gv; Figure 10) was not. The latter agrees with the failure to detect any changes in astrocyte HCs following INI-0602 administration *in vivo*, since Gv is more linked with HC activity than Gm [41]. Another beneficial effect of INI-0602 was its ability to modify resting membrane potential in CLN3^Δex7/8^ astrocytes. Although HC opening in various brain regions of CLN3^Δex7/8^ mice was only transient, it is clear that INI-0602 exerts beneficial effects, which may be explained, in part, by its ability to enhance astrocyte GJC and modify membrane conductance and potential. We found that INI-0602 blocked HC opening in acute brain slices from untreated mice, whereas, surprisingly, no changes in HC activity were observed in slices recovered from animals after a one month treatment period with INI-0602 *in vivo*. This discrepancy could be explained by the short-term actions of INI-0602 in the CNS. Indeed, Takeuchi et. al. reported a steady decline in brain INI-0602 concentrations *in vivo* during the first three hours after injection when the drug was administered to mice every other day, as was done in the current study [68]. In addition, our *in vitro* studies in acute brain slices showed that INI-0602 was easily washed out of brain tissues and, as such, its primary effect (i.e. inhibition of HC activity) is expected to be relatively short-lived. Therefore, the repetitive inhibition of astrocyte HC activity and/or membrane depolarization during the one month treatment period with INI-0602 may cause secondary adaptations in the brains of CLN3^Δex7/8^ mice, resulting in the therapeutic benefits reported here (i.e. reducing lysosomal inclusion burdens). This possibility is supported by the fact that astrocyte GJC was significantly increased following INI-0602 treatment, which, theoretically would occur by HC pairing to form more GJs and/or HC closure. Another option is that INI-0602 may influence pathways that are distinct from HC/GJ. This is suggested by the fact that CBX, the parental compound from which INI-0602 was derived, possesses other effects besides regulating GJ/HC activity [78], [79], [80] and was only partially capable of blocking INI-0602 action in acute brain slices (Figure 8B). Indeed, the changes in astrocytic membrane potential and conductance evoked by INI-0602 in brain slices are expected to influence other cellular functions in the CNS. An alternative explanation is that INI-0602 changes membrane fluidity and/or alters the trafficking patterns of molecules that improve disease outcomes, attributes that are consistent with other GJ/HC inhibitors [81], [82]. By extension, INI-0602 may slow HC diffusion in the lipid bilayer to facilitate the docking of HCs on neighboring astrocytes and/or promote HC trafficking to the plasma membrane, both of which would facilitate the establishment of GJ channels. This possibility agrees with our findings that astrocyte GJC was significantly increased in both WT and CLN3^Δex7/8^ astrocytes following a one month treatment period with INI-0602; however, future studies outside the scope of the current report are needed to support this mechanism of action. A final option emanates from the finding that CLN3^Δex7/8^ astrocyte HC activity declines over time. Since mice were 60 days-old following the one month dosing period with INI-0602, the natural HC closure observed in untreated CLN3^Δex7/8^ mice could account for the inability to detect a major effect with INI-0602 treatment, since a large majority of HCs were already closed. However, the fact that INI-0602 significantly increased GJC in both CLN3^Δex7/8^ and WT astrocytes implies that the drug was still closing HCs, albeit at a level that was not detectable by our *ex vivo* methodology. Although INI-0602 significantly reduced inclusion burdens CLN3^Δex7/8^ mice it did not show any clear effects on glutamate or GABA levels as measured by MR spectroscopy, which may originate from the dosing regimen and/or drug concentration used, age of the animals, and/or length of time mice received the compound. Experiments are ongoing to determine whether long-term INI-0602 treatment can limit neuronal death observed at advanced ages in CLN3^Δex7/8^ animals (i.e. 6–8 months).

Previous work from us and others has shown a reciprocal relationship between GJC and HC activity in the context of injury or inflammation [41], [42]. In general, pathological disturbances lead to reduced astrocyte GJC and a concomitant opening of HCs, which is thought to be attributed, in part, to the action of proinflammatory mediators [25], [26]. In the current report, increased HC activity was observed in the S1C of CLN3^Δex7/8^ mice at postnatal day 30, which correlated with a trend towards decreased GJC in the same region. Likewise, HC activity was significantly decreased in the HPC at postnatal day 90, which correlated with increased GJC in this same location. Nonetheless, it appears too simplistic to expect changes in GJC to always parallel HC function, particularly given the vast differences in cell types and complexity in various brain regions. We propose that the increase in GJC observed in CLN3^Δex7/8^ mice may represent a compensatory response to failing astrocyte “health” as demonstrated by the decreased glutamine synthetase and GLAST expression reported here in the face of elevated glutamate levels in the JNCL brain as described by other groups [53], [59], [61]. Importantly, we found that GFAP expression was significantly elevated in CLN3^Δex7/8^ mice at postnatal day 90 in the VC, HPC, and TH in agreement with previous reports from other laboratories in CLN3 mutant mice [8], [20]. This is a key observation, since it indicated that the decreases in Cx43, GLAST, and glutamine synthetase detected in CLN3^Δex7/8^ animals did not result from astrocyte loss. Rather, these findings reveal the attrition of molecules that regulate glutamate homeostasis, which likely triggers astrocyte activation in an attempt to compensate for this decline. It remains to be determined whether these GFAP-reactive astrocytes exert a beneficial or detrimental response in the context of disease progression. It was surprising that Cx43 and Cx30 staining were both reduced at postnatal day 90, yet astrocyte GJC was unaffected. A potential explanation for this observation is that abundant Cx43 and Cx30 protein still remained, which was likely sufficient to maintain functional GJ channels, particularly since HC activity was dramatically decreased at postnatal day 90 in several brain regions of CLN3 mutant animals. Alternatively, it is possible that CLN3 mutation leads to a redistribution in Cx43 and/or Cx30 that favors GJ plaque formation rather than unopposed HCs. This could conceivably result from increased homing of Cxs to GJ plaques and/or impaired plaque turnover. In addition, astrocytes have been reported to express Cx26 [83], which could compensate for the reductions in Cx43 and Cx30 observed in CLN3^Δex7/8^ animals. Finally, pannexin proteins are also expressed by astrocytes and form HCs [84], [85]. It is possible that pannexin levels are altered in CLN3^Δex7/8^ mice, which may account for some of the differences in HC activity observed in the current study. The reduction in Cx43 expression was congruent with decreases in glutamine synthetase and GLAST levels in CLN3^Δex7/8^ mice that were more profound in comparison, suggestive of a generalized decline in astrocyte homeostasis. Future studies examining these parameters in older CLN3^Δex7/8^ animals will reveal whether these changes are progressive and, if so, they could conceivably augment neuronal pathology and eventual cell death.

In conclusion, our study has identified a novel decline in astrocyte function over the first three postnatal months in CLN3^Δex7/8^ mice. Impairments in astrocyte homeostatic effects, as suggested by the dramatic reduction in glutamine synthetase and GLAST expression in CLN3^Δex7/8^ animals, can compromise neuronal function and conceivably impact neuronal demise during JNCL. We also observed widespread opening of astrocyte HCs in numerous brain regions, which although transient, could set the stage for downstream pathological effects. Indeed, targeting early changes in astrocyte HC function in the CLN3^Δex7/8^ brain with the CBX derivative INI-0602 led to reductions in lysosomal ceroid inclusions. The exact mechanisms whereby INI-0602 provides these beneficial effects remain to be elucidated; however, its ability to enhance astrocyte GJC and modify membrane properties are likely candidates based on the ability of astrocyte syncytia to detoxify glutamate within the CNS. Nevertheless, INI-0602 appears to represent an attractive candidate for further development in the context of JNCL therapeutics.

## Materials and Methods

### Ethics Statement

This study was conducted in strict accordance with the recommendations in the Guide for the Care and Use of Laboratory Animals of the National Institutes of Health. The protocol was approved by the Institutional Animal Care and Use Committee of the University of Nebraska Medical Center (Approval ID: 11-074-08-EP).

### Mice

CLN3^Δex7/8^ mice (C57BL/6 background) that lack a 1.02 kb segment spanning exons 7 and 8 of *CLN3* were used [52]. This represents the most common mutation in ∼85% JNCL patients and CLN3^Δex7/8^ mice phenocopy several aspects of JNCL, including neuronal loss, glial activation, metabolic disturbances, and progressive storage material deposition [4], [52], [86], [87]. Age- and sex-matched C57BL/6 mice were used as WT controls (The Jackson Laboratory, Bar Harbor, ME). Both male and female mice were utilized for these studies and no gender influences on experimental outcomes were observed. To analyze early pathological changes during JNCL, CLN3^Δex7/8^ and WT mice (n = 54 and 48, respectively) were evaluated at postnatal days 30–37, 60–67, and 90–97 (referred to throughout the study as postnatal days 30, 60, and 90, respectively). In experiments to quantitate ceroid inclusions, tissues from 12–16 month-old mice were included for comparisons with younger animals.

In some experiments, GFAP-GFP transgenic mice, where GFP expression is driven by the human GFAP promoter (8–12 weeks of age; The Jackson Laboratory) [88], were used to confirm astrocyte staining specificity with CellTracker Blue CMAC (CTB; Invitrogen, San Diego, CA).

### *In vivo* Administration of the HC Inhibitor INI-0602

In some experiments, CLN3^Δex7/8^ and WT mice were treated with the blood-brain barrier permeable CBX analog INI-0602 [68] to determine the functional importance of astrocyte HC activity in disease pathogenesis. CLN3^Δex7/8^ and WT mice received i.p. injections of PBS or 10 mg/kg INI-0602 every other day from postnatal days 30 to 60 (n = 6–17 per group), a dosing regimen that was optimal at reducing clinical symptoms in mouse models of ALS and AD [68]. At the end of the 30 day treatment period, mice were subjected to magnetic resonance (MR) spectroscopy to quantitate the brain metabolome, whereupon they were sacrificed and tissues utilized for subsequent studies. Blood was collected from CLN3^Δex7/8^ and WT mice treated with INI-0602 or vehicle upon sacrifice, whereupon blood chemistry profiles were evaluated using a VetScan2 blood analyzer (Abaxis, Union City, CA; Comprehensive Diagnostic Profile rotor #500-1038).

### Magnetic Resonance (MR) Spectroscopy

To evaluate the impact of INI-0602 treatment on the CNS metabolome, MR spectroscopy scanning was conducted on CLN3^Δex7/8^ and WT mice following the one month INI-0602 dosing interval. Briefly, animals were anesthetized with 1.5% isoflurane in a 70% nitrous oxide/30% oxygen mixture and positioned in a custom-made stereotactic holder equipped with a MRI-compatible physiological monitoring system (Model 1025, SA Instruments, Stony Brook, NY). Core body temperature of anesthetized animals was maintained by a warm air delivery system and MR spectroscopy data was obtained using a Bruker Avance 7 Tesla/21 cm small bore system (Billerica, MA) and a lab-built birdcage coil designed for mouse brain imaging. The initial setup for magnetic resonance imaging (MRI) studies included a 3-plane locator scan, where localization and initial shimming using a novel field mapping method was utilized [89]. Once the mouse was positioned and shimmed, MR spectroscopy spectra were obtained from the HPC of CLN3^Δex7/8^ and WT mice±INI-0602 treatment. MR spectroscopy was acquired using 576 averages with a repetition time of 4000 ms, echo time of 50 ms, and 3 kHz bandwidth. Results from quality assurance (QA) phantoms were used to verify accuracy and random error measurements in mice.

### Spectroscopic Processing and Analyses

Spectroscopic data were processed by removal of residual water signal using the HLSVD filter. Spectra from ^1^H MR spectroscopy data sets were curve fit in the time domain using the QUantitation based on Quantum ESTimation (QUEST) algorithm [90], [91] in jMRUI (Java-based version of the magnetic resonance user interface (MRUI) quantitation package), which fits results to a sum of individual metabolite spectra (basis set). Spectra for the basis set were acquired from QA phantoms with the same acquisition parameters used *in vivo*. Phantoms containing either alanine, aspartate, choline, creatine, gamma-amino butyric acid, glutamate, glutamine, glycerophosphocholine, glycine, lactate, myo-inositol, NAA, or taurine were maintained at 38°C using a circulating water bath and buffered to pH 7.4 at physiological osmolarity. Results were normalized to the sum of all 13 metabolites as a semi-quantitative method for reporting metabolite concentrations.

### Preparation of Acute Brain Slices and SR101/CTB Staining

To prepare acute brain slices for quantitating astrocyte GJ/HC activity and electrophysiological parameters, CLN3^Δex7/8^ and WT mice were decapitated, whereupon the brains were quickly removed and bathed in ice-cold artificial cerebrospinal fluid (ACSF, in mM: 124 NaCl, 26 NaHCO_3_, 3 KCl, 2 MgCl_2_, 2 CaCl_2_, 0.4 ascorbic acid, 10 glucose) continuously bubbled with carbogen (95% O_2_ and 5% CO_2_) and maintained at pH, 7.4. Next, 400 µm thick coronal slices were prepared using a Leica VT1000S vibrating-blade microtome (Leica Microsystems, Germany) and immediately placed in ACSF at 32°C. During a 20–30 min incubation period at 32°C, some slices were stained with the astrocyte-selective dye SR101 (400 nM, Sigma-Aldrich, St. Louis, MO) [48] and held in ACSF at room temperature for at least 1 h before use. Other slices were stained with the fluorescent dye CellTracker Blue CMAC (2 µM; Invitrogen) for 30 min in ACSF at room temperature and immediately used for experiments assessing astrocyte HC activity. Brain slices maintained good viability for a 6–8 h period, an interval that was not exceeded in the current study, as demonstrated by stable electrophysiological properties in patched astrocytes across the recording intervals examined. In addition, the CTB dye utilized in these studies was specifically designed to stain live, but not dead cells. By extension, the robust CTB staining reported here provides independent confirmation of cell viability in our acute brain slices. Upon completion of some experiments, CTB-stained brain slices were fixed in 10% formaldehyde and processed according to the histological protocol described below for clarifying the identity of CTB^+^ cells.

### Brain Regions Examined

To investigate region-dependent changes in astrocyte function in CLN3^Δex7/8^ mice, five brain regions were selected for analysis based on prior reports documenting significant pathology in older animals [6], [8], [20], [52]. These included, layers IV-VI of the primary somatosensory cortex (S1C); dorsal striatum (STR); layers IV-VI of the primary and secondary areas of the visual cortex (VC); stratum radiatum layer in the CA1 field of the hippocampus (HPC); and ventral posterolateral and posteromedial nuclei of the thalamus (TH). These regions were identified using a mouse brain atlas [92].

### Quantification of HC Activity

For assessing astrocyte HC activity, acute brain slices from CLN3^Δex7/8^ and WT mice were pre-stained with CTB to facilitate astrocyte identification and incubated in a submerged chamber while constantly perfused with the HC-permeable dye EtBr (2.5 µM) in ACSF at a rate 1.5 ml/min at 30°C. Z-stack images of CTB and EtBr staining were collected at each of the five brain regions, with 3 random fields of view (FOV) examined for each brain structure. Images were captured at each location every 10 min before EtBr bath application and throughout the 30 min EtBr incubation period. Each image covered a FOV of 167×220 µm and a tissue depth of 55 µm with a total magnification of 400X. Astrocytes were identified by CTB staining with cell bodies marked as regions of interest (ROI), and an average EtBr intensity for each ROI was obtained (designated F) in the same Z-stack where the maximum intensity for CTB was found. Next, EtBr intensity was normalized to background values (F_0_) to account for autofluorescent ceroid inclusions, which were taken as the minimal densitometric mean value in the ROI Z-stack, according to the equation: (F-F_0_)/F_0_*100%. Astrocyte HC activity was calculated as the rate of EtBr uptake in arbitrary units per min (a.u./min) using a linear regression algorithm as previously described [41]. To determine the effect of INI-0602 on astrocyte HC activity and electrophysiological parameters in acute brain slices of CLN3^Δex7/8^ and WT mice, 100 µM INI-0602 was applied to the bath solution during EtBr uptake and electrophysiological measurements.

### Electrophysiology

Electrophysiological recordings of astrocytes were performed in the S1C and HPC of acute brain slices from CLN3^Δex7/8^ and WT mice (n = 29/group) as previously described [93]. Acute brain slices were incubated with SR101 to facilitate astrocyte identification. SR101 was utilized in these experiments instead of CTB, since the latter would interfere with AlexaFluor 350 contained in the recording electrode for visualization of astrocyte GJC. Recording electrodes were filled with a solution containing (in mM): 110 K-gluconate, 20 KCI, 0.2 CaCl_2,_ 1 MgCl_2_, 5 EGTA, and 10 HEPES, pH 7.4 with an electrical resistance of 8–10 MΩ. Whole-cell patch-clamp recordings were performed on astrocytes using a computer-controlled amplifier (Multiclamp 700B, Axon Instruments/Molecular Devices Corp., Sunnyvale, CA) and a video setup. The membrane resistance (Rm) and access resistance (Ra) of astrocytes were calculated using the membrane test function in pClamp-10 (Axon Instruments/Molecular Devices). Resting input conductance (Gm) was calculated by the formula, Gm = Gt *[Rt/Rm], where Rt is total resistance (Rm+Ra) and Gt is total conductance calculated as the linear slope coefficient of the voltage-current relationship (I–V) near the resting membrane potential (RMP, zero holding current). Using this method, cell input conductance (Gi) was calculated at every command voltage (Vc) (on average, from −140 to +60 mV with 5 mV step). All Gi points obtained from 3–4 primary I–V recordings within the Vc range from −90 to −50 mV and RMP range from −90 to −60 mV were included for statistical analysis to evaluate Gm. As observed here and in prior studies, Gi fluctuated over the voltage range and Gi deviation can be associated with voltage-dependent conductance (Gv). In the current report, Gv plot area was calculated using the surveyor’s formula for the area of a simple polygon utilizing maximal and minimal Gi points within the Vc range from −90 to 0 mV as determined by the equation:
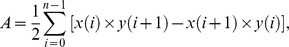
where x_i_ = Vc (mV) and y_i_ = [Gi - minimal Gi] (nS) on voltage step i from 0 to n-1; A - Gv plot area as the sum of absolute values (mV*nS or pA). To estimate Gv, the 4 maximal [A] values found within 10 consequential I-V recordings were included in the calculation from each recorded cell. Gv should be largely independent from Ra, since membrane potential and conductance used in the formula are oppositely modified in response to Ra changes, as can be concluded from the I-V relationship.

### Quantitation of Astrocyte GJC

The GJ permeable dye AlexaFluor 350 (0.5 mM, Invitrogen, San Diego, CA) was included in the patch pipette to visualize the degree of GJC in astrocytes from the S1C or HPC in acute brain slices from CLN3^Δex7/8^ and WT mice after 15–20 min of whole-cell patch-clamp recording. Calculations of astrocyte GJC were performed by enumerating the number of superimposed cell images with AlexaFluor 350 and SR101 fluorescence using appropriate filters in a microscopic FOV of 334×448 µm with a total magnification of 200X. Cell coupling was confirmed by the quantitation of fluorescent intensities using AxioVision software (Zeiss, Germany). Cells were considered GJ coupled if the peak optical intensities for AlexaFluor 350 exceeded 10% of background levels.

### Quantitative Measurements of Immunofluorescence Staining and Ceroid Inclusions

For histological analysis, brain slices from CLN3^Δex7/8^ and WT mice were fixed in 10% formaldehyde for 1–2 h, washed in PBS (pH = 7.4), cryoprotected with 30% sucrose overnight, and fast-frozen in OCT embedding medium. Next, 20 µm thick cryostat sections were mounted onto glass slides, air dried, and stored at −20°C until use. Cx43, Cx30, glutamine synthetase, GLAST, and GFAP expression were evaluated in the same five brain regions as HC studies by immunofluorescence staining. Primary antibodies for Cx43 and Cx30 (Molecular Probes/Life Technologies, Carlsbad, CA; #71-0700 and #71-2200, respectively) and glutamine synthetase and GLAST (Abcam, Cambridge, MA; #AB16802 and #AB416, respectively) were visualized using a fluorescein-conjugated secondary antibody (Jackson Immunoresearch Laboratories, West Grove, PA), whereas GFAP (Dako, #20334), MAP2 (Chemicon, Temecula, CA, #AB5622), and Iba-1 (Biocare Medical, #Cp290B) were detected using a biotin-streptavidin approach. Quenching of autofluorescent inclusions in CLN3^Δex7/8^ tissues was achieved by incubating slides with a 70% solution of Sudan Black for 10 min (Figure S2). Tissue sections were imaged using a Carl Zeiss LSM 510 META confocal microscope in a FOV 450×450 µm (200× magnification) or 225×225 µm FOV (400× magnification). Cx43, Cx30, glutamine synthetase, GLAST, and GFAP expression were quantitated using AxioVision software (Zeiss) as the mean intensity staining values (F) normalized to background values in tissue sections where primary antibodies were omitted (F_bkgr_), i.e. F/F_bkgr_.

For quantitating autofluorescent ceroid inclusions, images were acquired from live brain slices for each of the five brain regions examined utilizing a GFP filter set (38 HE GFP, EX 470, EM 525; Zeiss). Each image covered a FOV of 167×220 µm and a tissue depth of 20 µm. Quantitation was performed using AxioVision software with results reported as the area of autofluorescent inclusions (µm^2^) for each image.

### Western Blot Analysis

Coronal sections (1200 µm thick) were prepared from acute brain slices using a vibratome while bathed in ice-cold ACSF, whereupon the five brain regions described above were manually dissected to collect total protein extracts. Tissues were homogenized in cell lysis buffer [50 mM Tris-HCl, pH 7.5; 150 mM sodium chloride; 0.5% Triton X-100; 1 mM sodium orthovanadate; 10 mM sodium fluoride; 0.5 mM phenylmethanesulfonyl fluoride and supplemented with complete protease inhibitor (Roche, South San Francisco, CA) and phosphatase inhibitor (Thermo Scientific, Waltham, MA) tablets]. Twenty µg of total protein was run on 10% PAGE gels, whereupon Western blotting was performed as previously described [94]. Blots were probed with Cx43 and Cx30 (both from Molecular Probes) and glutamine synthetase and GLAST antibodies (both from Abcam) and developed by chemiluminescence. Blots were stripped and re-probed with an antibody against β-actin (Sigma, St. Louis, MO) to confirm uniformity in gel loading. Blots were quantitated by densitometry analysis using an Alpha Innotech imager (Protein Simple, San Jose, CA), where signals were normalized to β-actin.

### Statistical Analyses

A Student’s two-tailed *t*-test was used for data analyses (MS Excel 2007), with values reported as the mean ± SEM compiled from independent experiments.

## Supporting Information

Figure S1 **Age-dependent changes in astrocyte gap junction (GJ) communication in CLN3^Δex7/8^ mice.** Acute brain slices were prepared from wild type (WT) and CLN3^Δex7/8^ mice at postnatal days 30, 60 and 90, whereupon the number of GJ coupled astrocytes was evaluated in the somatosensory cortex (S1C) and hippocampus (HPC) by monitoring the passage of the GJ permeable dye AlexaFluor 350 injected in a single astrocyte after whole-cell patch clamp recordings (n = 16–41 astrocytes per group). Significant differences between WT and CLN3^Δex7/8^ astrocytes are denoted by asterisks (*p<0.05).
(TIF)

Figure S2 **Sudan black quenches the autofluorescence of lysosomal ceroid inclusions in CLN3^Δex7/8^ mice.** Cryostat sections (20 µm) were prepared from the somatosensory cortex (S1C) and hippocampus (HPC) of 14 month-old CLN3^Δex7/8^ mice to visualize robust inclusion deposition across multiple wavelengths. Sections were incubated with 10% Sudan Black for 10 min followed by DAPI staining to visualize nuclei.
(TIF)

Figure S3 **Connexin 43 (Cx43) expression is differentially regulated in various brain regions of CLN3^Δex7/8^ mice.** Total protein extracts were prepared from the somatosensory cortex (S1C), visual cortex (VC), hippocampus (HPC), striatum (STR), and thalamus (TH) of wild type (WT) and CLN3^Δex7/8^ mice (n = 3–4/group), whereupon samples were analyzed by Western blotting for Cx43. Each blot was stripped and re-probed for β-actin to assess uniformity in gel loading. Results are presented as (A) raw data from the S1C and VC and (B) quantitation following β-actin normalization. Significant differences between WT and CLN3^Δex7/8^ tissues are denoted by asterisks (*p<0.05; **p<0.01).
(TIF)

Figure S4 **Glutamine synthetase (GS) expression is reduced in select brain regions of CLN3^Δex7/8^ mice.** Total protein extracts were prepared from the somatosensory cortex (S1C), visual cortex (VC), hippocampus (HPC), striatum (STR), and thalamus (TH) of wild type (WT) and CLN3^Δex7/8^ mice (n = 3–4/group), whereupon samples were analyzed by Western blotting for glutamine synthetase (GS). Each blot was stripped and re-probed for β-actin to assess uniformity in gel loading. Results are presented as (A) raw data from the S1C and VC and (B) quantitation following β-actin normalization. Significant differences between WT and CLN3^Δex7/8^ tissues are denoted by asterisks (*p<0.05; **p<0.01).
(TIF)

Figure S5 **Connexin 30 expression in various brain regions of CLN3^Δex7/8^ mice.** Total protein extracts were prepared from the somatosensory cortex (S1C), visual cortex (VC), and hippocampus (HPC) of wild type (WT) and CLN3^Δex7/8^ mice (n = 3–4/group), whereupon samples were analyzed by Western blotting for Cx30. Each blot was stripped and re-probed for β-actin to assess uniformity in gel loading. Results are presented as (A) raw data from the S1C and VC and (B) quantitation following β-actin normalization.
(TIF)

Figure S6 **GLAST expression in various brain regions of CLN3^Δex7/8^ mice.** Total protein extracts were prepared from the somatosensory cortex (S1C), visual cortex (VC), and hippocampus (HPC) of wild type (WT) and CLN3^Δex7/8^ mice (n = 3–4/group), whereupon samples were analyzed by Western blotting for GLAST. Each blot was stripped and re-probed for β-actin to assess uniformity in gel loading. Results are presented as (A) raw data from the S1C and VC and (B) quantitation following β-actin normalization.
(TIF)

Figure S7 ***In vivo*** **administration of INI-0602 does not alter HC activity in CLN3^Δex7/8^ mice.** CLN3^Δex7/8^ and wild type (WT) mice were treated with INI-0602 (10 mg/kg) or PBS from postnatal day 30 to 60, whereupon EtBr uptake was measured in CTB stained astrocytes in the somatosensory cortex (S1C), visual cortex (VC), hippocampus (HPC), striatum (STR), and thalamus (TH) as a quantitative measure of HC activity, which is expressed in arbitrary units (a.u.) per min (n = 9 mice/group). Significant differences between WT and CLN3^Δex7/8^ mice are indicated by asterisks (*p<0.05, **p<0.01, ***p<0.001).
(TIF)

Table S1 **Electrophysiological parameters of cortical astrocytes in CLN3^Δex7/8^ and WT mice.**
(DOCX)

Table S2 **Electrophysiological parameters of hippocampal astrocytes in CLN3^Δex7/8^ and wild type (WT) mice.**
(DOCX)

Table S3 ***In vivo*** **administration of INI-0602 does not alter blood chemistry profiles in either WT or CLN3^Δex7/8^ mice.**
(DOCX)
