# Supplementary figures and images for: Evidence for Aberrant Astrocyte Hemichannel Activity in Juvenile Neuronal Ceroid Lipofuscinosis (JNCL)
Source: PLoS One. 2014 Apr 15;9(4):e95023. doi: 10.1371/journal.pone.0095023 (PMC3988164; doi:10.1371/journal.pone.0095023)

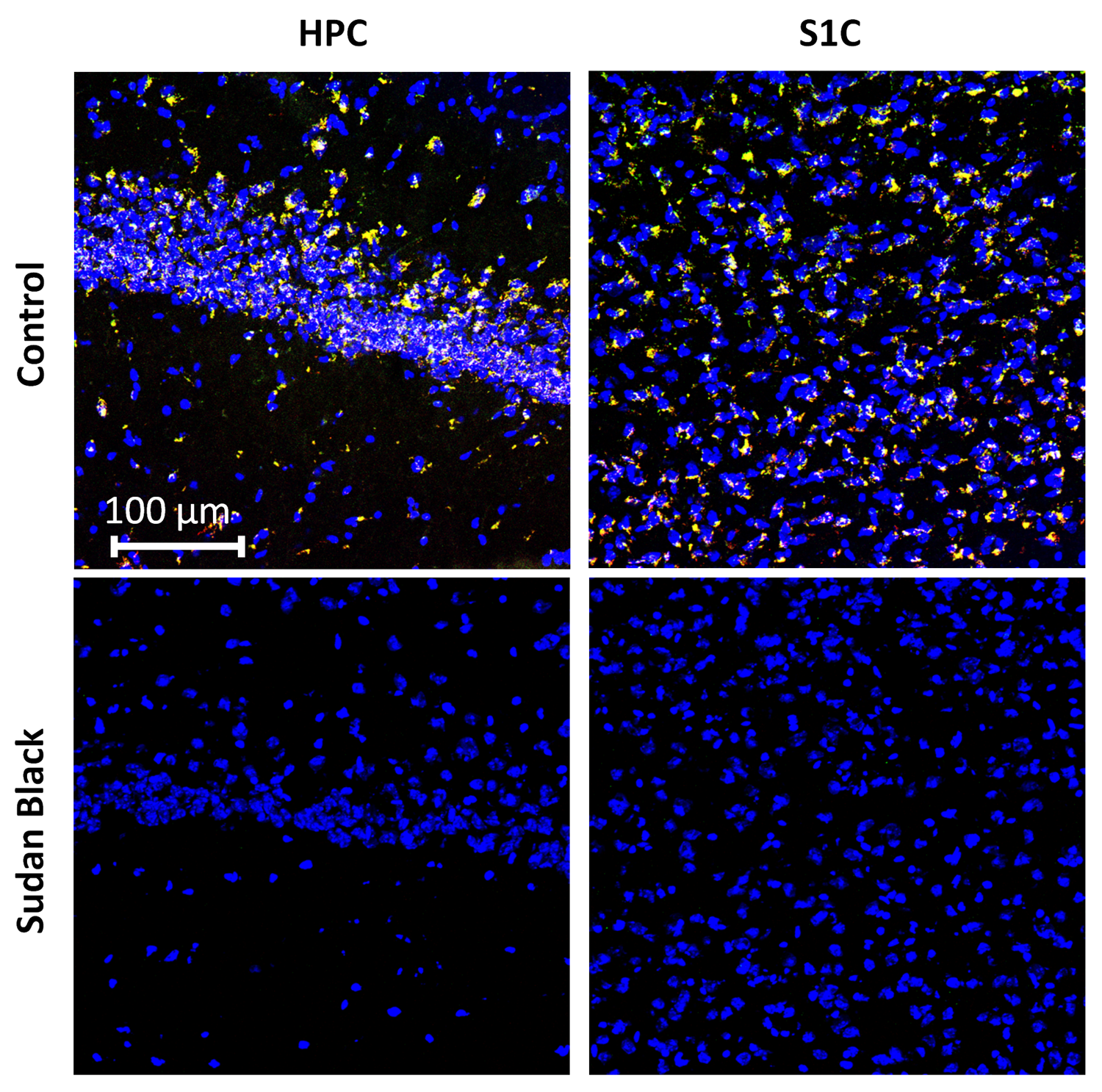

Supplement: Figure S2 — Sudan black quenches the autofluorescence of lysosomal ceroid inclusions in CLN3Δex7/8 mice. Cryostat sections (20 µm) were prepared from the somatosensory cortex (S1C) and hippocampus (HPC) of 14 month-old CLN3Δex7/8 mice to visualize robust inclusion deposition across multiple wavelengths. Sections were incubated with 10% Sudan Black for 10 min followed by DAPI staining to visualize nuclei. (TIF) [file pone.0095023.s002.tif]

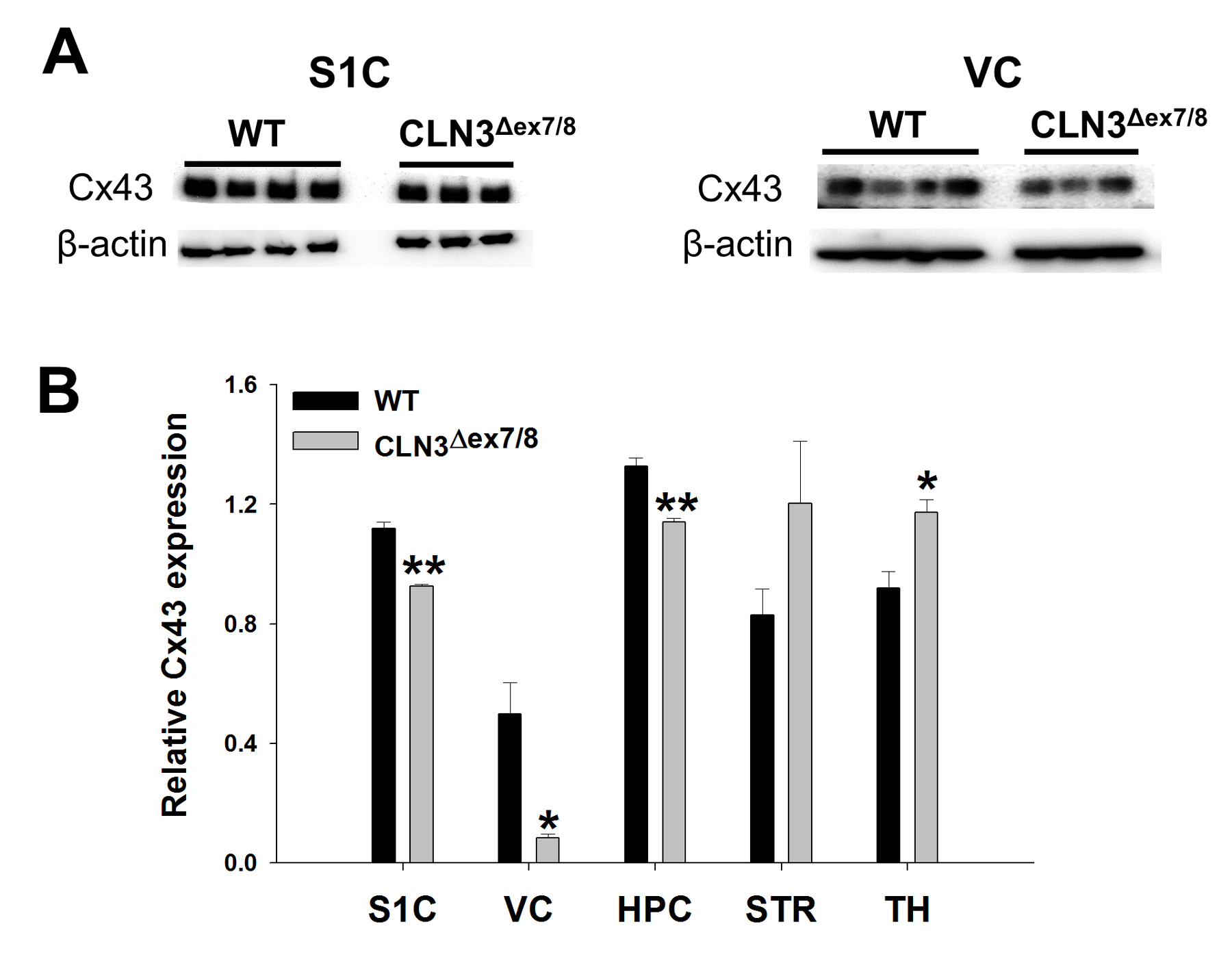

Supplement: Figure S3 — Connexin 43 (Cx43) expression is differentially regulated in various brain regions of CLN3Δex7/8 mice. Total protein extracts were prepared from the somatosensory cortex (S1C), visual cortex (VC), hippocampus (HPC), striatum (STR), and thalamus (TH) of wild type (WT) and CLN3Δex7/8 mice (n = 3–4/group), whereupon samples were analyzed by Western blotting for Cx43. Each blot was stripped and re-probed for β-actin to assess uniformity in gel loading. Results are presented as (A) raw data from the S1C and VC and (B) quantitation following β-actin normalization. Significant differences between WT and CLN3Δex7/8 tissues are denoted by asterisks (*p<0.05; **p<0.01). (TIF) [file pone.0095023.s003.tif]

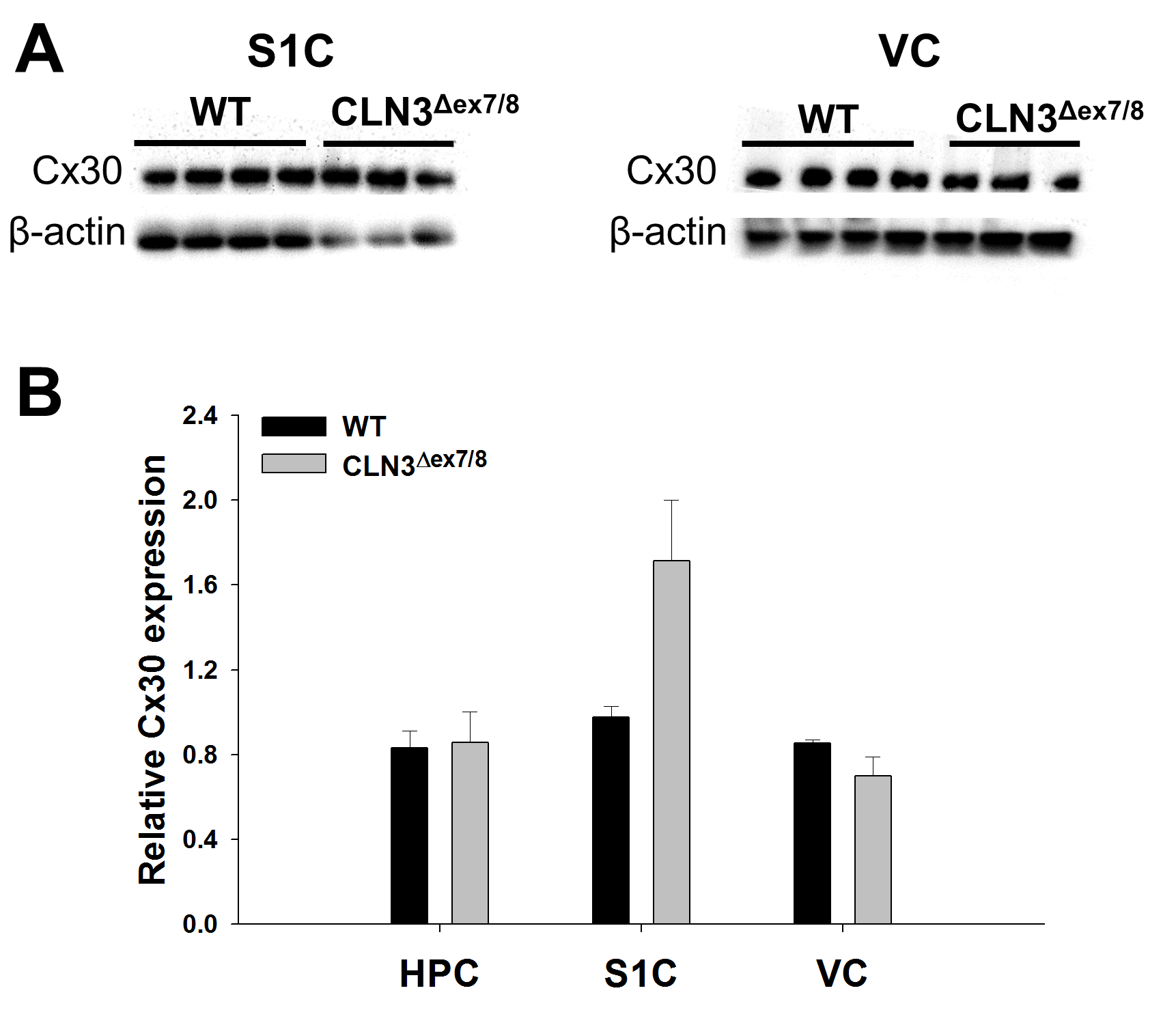

Supplement: Figure S4 — Glutamine synthetase (GS) expression is reduced in select brain regions of CLN3Δex7/8 mice. Total protein extracts were prepared from the somatosensory cortex (S1C), visual cortex (VC), hippocampus (HPC), striatum (STR), and thalamus (TH) of wild type (WT) and CLN3Δex7/8 mice (n = 3–4/group), whereupon samples were analyzed by Western blotting for glutamine synthetase (GS). Each blot was stripped and re-probed for β-actin to assess uniformity in gel loading. Results are presented as (A) raw data from the S1C and VC and (B) quantitation following β-actin normalization. Significant differences between WT and CLN3Δex7/8 tissues are denoted by asterisks (*p<0.05; **p<0.01). (TIF) [file pone.0095023.s004.tif]

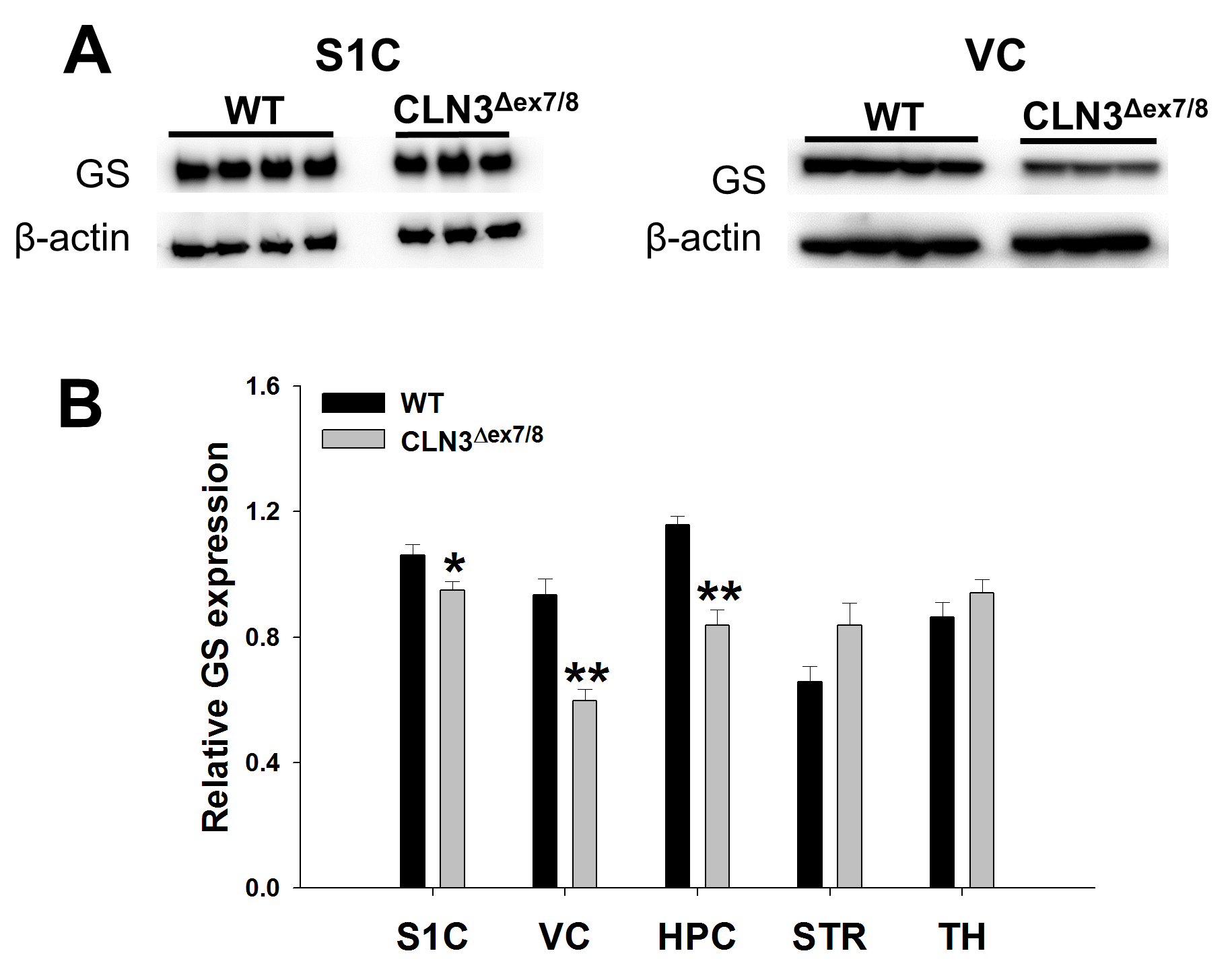

Supplement: Figure S5 — Connexin 30 expression in various brain regions of CLN3Δex7/8 mice. Total protein extracts were prepared from the somatosensory cortex (S1C), visual cortex (VC), and hippocampus (HPC) of wild type (WT) and CLN3Δex7/8 mice (n = 3–4/group), whereupon samples were analyzed by Western blotting for Cx30. Each blot was stripped and re-probed for β-actin to assess uniformity in gel loading. Results are presented as (A) raw data from the S1C and VC and (B) quantitation following β-actin normalization. (TIF) [file pone.0095023.s005.tif]

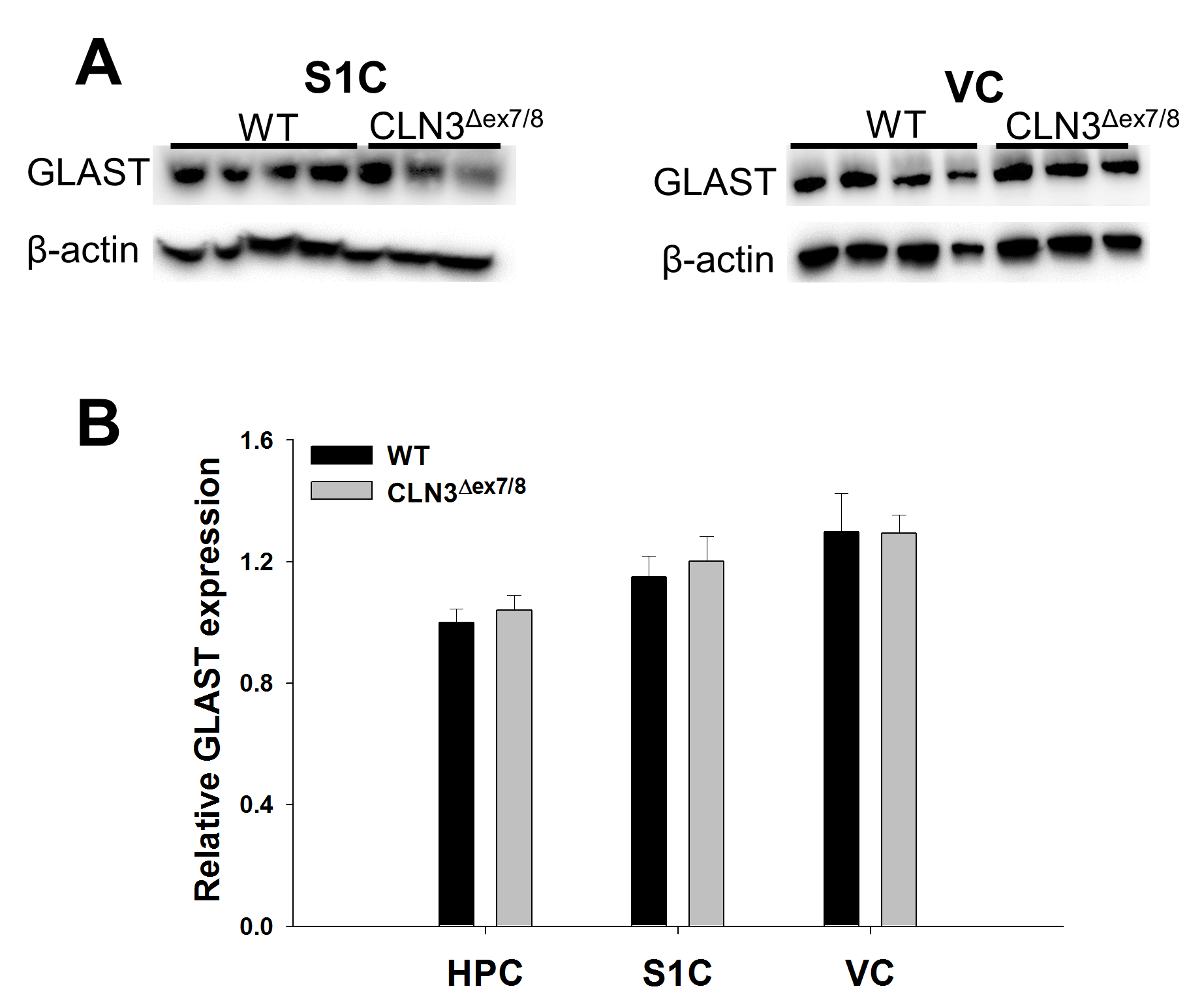

Supplement: Figure S6 — GLAST expression in various brain regions of CLN3Δex7/8 mice. Total protein extracts were prepared from the somatosensory cortex (S1C), visual cortex (VC), and hippocampus (HPC) of wild type (WT) and CLN3Δex7/8 mice (n = 3–4/group), whereupon samples were analyzed by Western blotting for GLAST. Each blot was stripped and re-probed for β-actin to assess uniformity in gel loading. Results are presented as (A) raw data from the S1C and VC and (B) quantitation following β-actin normalization. (TIF) [file pone.0095023.s006.tif]
