# Supplementary material for: Evidence for Aberrant Astrocyte Hemichannel Activity in Juvenile Neuronal Ceroid Lipofuscinosis (JNCL)
Source: PLoS One. 2014 Apr 15;9(4):e95023. doi: 10.1371/journal.pone.0095023 (PMC3988164; doi:10.1371/journal.pone.0095023)
Supplement: Table S1 — Electrophysiological parameters of cortical astrocytes in CLN3Δex7/8 and WT mice. (DOCX) [file pone.0095023.s008.docx]

**Supplemental Table 1. Electrophysiological parameters of cortical astrocytes in CLN3^Δex7/8^ and WT mice**

|  | PN | RMP (mV) | N | Cells | Gm (nS) | N | Cells | Gv (pA) | N | Cells |
| --- | --- | --- | --- | --- | --- | --- | --- | --- | --- | --- |
| WT | 30 | -73.4 ± 0.4 | 88 | 18 | 599.6 ± 17.8 | 271 | 13 | 148.9 ± 15.1 | 69 | 18 |
|  | 60 | -72.0 ± 0.4 | 88 | 18 | 618.7 ± 12.5 | 582 | 17 | 167.8 ± 19.7 | 65 | 19 |
|  | 90 | -74.0 ± 0.3 | 137 | 24 | 532.0 ± 12.6 | 784 | 19 | 210.8 ± 13.4 | 166 | 42 |
| CLN3^Δex7/8^ | 30 | -73.4 ± 0.5 | 123 | 24 | 547.3 ± 9.2* | 615 | 15 | 121.6 ± 10.3 | 97 | 27 |
|  | 60 | -72.5 ± 0.5 | 80 | 14 | 532.5 ± 12.9* | 388 | 11 | 120.3 ± 10.4* | 52 | 14 |
|  | 90 | -75.4 ± 0.3* | 145 | 27 | 472.2 ± 9.1* | 829 | 23 | 175.2 ± 10.2* | 163 | 41 |

Asterisks represent significant differences between wild type (WT) and CLN3^Δex7/8^ mice (*, *p* < 0.05); PN, postnatal day; RMP, resting membrane potential; Gm, resting membrane conductance; Gv, voltage-dependent membrane conductance
