# Supplementary material for: Evidence for Aberrant Astrocyte Hemichannel Activity in Juvenile Neuronal Ceroid Lipofuscinosis (JNCL)
Source: PLoS One. 2014 Apr 15;9(4):e95023. doi: 10.1371/journal.pone.0095023 (PMC3988164; doi:10.1371/journal.pone.0095023)
Supplement: Table S2 — Electrophysiological parameters of hippocampal astrocytes in CLN3Δex7/8 and wild type (WT) mice. (DOCX) [file pone.0095023.s009.docx]

**Supplemental Table 2. Electrophysiological parameters of hippocampal astrocytes in CLN3^Δex7/8^ and wild type (WT) mice**

|  | PN | RMP (mV) | N | Cells | Gm (nS) | N | Cells | Gv (pA) | N | Cells |
| --- | --- | --- | --- | --- | --- | --- | --- | --- | --- | --- |
| WT | 30 | -74.3 ± 1.1 | 39 | 13 | 584.6 ± 14.6 | 112 | 5 | 98.1 ± 23.4 | 36 | 12 |
|  | 60 | -74.4 ± 0.4 | 87 | 14 | 497.0 ± 12.8 | 482 | 12 | 118.7 ± 13.8 | 55 | 14 |
|  | 90 | -75.7 ± 0.5 | 104 | 15 | 511.8 ± 10.1 | 750 | 14 | 153.8 ± 19.6 | 56 | 14 |
| CLN3^Δex7/8^ | 30 | -76.1 ± 1.0 | 40 | 10 | 561.1 ± 21.4 | 200 | 5 | 111.7 ± 34.5 | 30 | 9 |
|  | 60 | -76.5 ± 0.4* | 150 | 22 | 436.1 ± 8.6* | 719 | 15 | 113.4 ± 13.7 | 83 | 21 |
|  | 90 | -76.3 ± 0.3 | 134 | 18 | 429.7 ± 5.4* | 824 | 15 | 105.5 ± 7.9* | 72 | 18 |

Asterisks represent significant differences between wild type (WT) and CLN3^Δex7/8^ mice (*, *p* < 0.05); PN, postnatal day; RMP, resting membrane potential; Gm, resting membrane conductance; Gv, voltage-dependent membrane conductance
