# Supplementary material for: Evidence for Aberrant Astrocyte Hemichannel Activity in Juvenile Neuronal Ceroid Lipofuscinosis (JNCL)
Source: PLoS One. 2014 Apr 15;9(4):e95023. doi: 10.1371/journal.pone.0095023 (PMC3988164; doi:10.1371/journal.pone.0095023)
Supplement: Table S3 — In vivo administration of INI-0602 does not alter blood chemistry profiles in either WT or CLN3Δex7/8 mice. (DOCX) [file pone.0095023.s010.docx]

**Supplemental Table 3.** ***In vivo* administration of INI-0602 does not alter blood chemistry profiles in either WT or CLN3^Δex7/8^ mice**

|  | ALP  (u/L) | ALT  (u/L) | TBIL  (mg/dL) | BUN  (mg/dL) | Ca^2+^  (mg/dL) | PHOS  (mg/dL) | GLU  (mg/dL) | Na^+^  (mmol/L) | TP  (g/dL) |
| --- | --- | --- | --- | --- | --- | --- | --- | --- | --- |
| Normal range | 35-222 | 17-77 | 0-0.9 | 9-33 | 6-13 | 5.7-9.2 | 140-263 | 110-195 | 3.9-6.4 |
| WT-PBS, n=3 | 55.7 ± 30 | 50 ± 7 | 0.17 ± 0.1 | 24 ± 2 | 10.5 ± 0.1 | 10.8 ± 1.7 | 185 ± 36 | 151 ± 0.6 | 5.8 ± 0.2 |
| WT-INI, n=2 | 73 ± 55 | 51 ± 11 | 0.20 ± 0.1 | 29 ± 8 | 10.4 ± 0.1 | 9.2 ± 1.4 | 174 ± 27 | 151 ± 1.5 | 6.1 ± 0.3 |
| CLN3^Δex7/8^-PBS, n=2 | 83 ± 3 | 27 ± 1 | 0.25 ± 0.1 | 20 ± 1 | 9.9 ± 0.1* | 6.9 ± 1.0 | 187 ± 23 | 147 ± 0.5** | 6.0 ± 0.1 |
| CLN3^Δex7/8^-INI, n=3 | 57.3 ± 12 | 22 ± 3 | 0.15 ± 0.1 | 22 ± 2 | 9.8 ± 0.2 | 8.8 ± 0.5 | 151 ± 14 | 147 ± 1.7 | 5.8 ± 0.3 |

Asterisks represent values significantly different in CLN3^Δ7/8^ versus wild type (WT) mice (*, *p* < 0.05; **, *p* < 0.01); INI; INI-0602; PBS, phosphate buffered saline; ALP, alkaline phosphatase; ALT, alanine aminotransferase; TBIL, total bilirubin; BUN, blood urea nitrogen; PHOS, phosphorous; GLU, glucose; TP, total protein
